# A glycan-based approach to therapeutic angiogenesis

**DOI:** 10.1371/journal.pone.0182301

**Published:** 2017-08-01

**Authors:** Jie Shi Chua, Vy M. Tran, Mausam Kalita, Maritza V. Quintero, Orlando Antelope, Geethu Muruganandam, Yukio Saijoh, Balagurunathan Kuberan

**Affiliations:** 1 Department of Bioengineering, University of Utah, Salt Lake City, Utah, United States of America; 2 Department of Medicinal Chemistry, University of Utah, Salt Lake City, Utah, United States of America; 3 Department of Neurobiology and Anatomy, University of Utah, Salt Lake City, Utah, United States of America; 4 Department of Biology, University of Utah, Salt Lake City, Utah, United States of America; Thomas Jefferson University, UNITED STATES

## Abstract

Angiogenesis, the sprouting of new blood vessels from existing vasculature, involves multiple complex biological processes, and it is an essential step for hemostasis, tissue healing and regeneration. Angiogenesis stimulants can ameliorate human disease conditions including limb ischemia, chronic wounds, heart disease, and stroke. The current strategies to improve the bioavailability of pro-angiogenic growth factors, including VEGF and FGF2, have remained largely unsuccessful. This study demonstrates that small molecules, termed click-xylosides, can promote angiogenesis in the *in vitro* matrigel tube formation assay and the *ex ovo* chick chorioallantoic membrane assay, depending on their aglycone moieties. Xyloside treatment enhances network connectivity and cell survivability, thereby, maintaining the network structures on matrigel culture for an extended period of time. These effects were achieved via the secreted xyloside-primed glycosaminoglycans (GAG) chains that in part, act through an ERK1/2 mediated signaling pathway. Through the remodeling of GAGs in the extracellular matrix of endothelial cells, the glycan approach, involving xylosides, offers great potential to effectively promote therapeutic angiogenesis.

## 1 Introduction

Angiogenesis, the formation of new blood vessels from existing ones, is an essential biological process during development for organogenesis and wound repair. In the embryo, the vasculature development is the initial sentinel event that provides nutrients, gas circulation, and instructive signals for organ morphogenesis [1]. Upon reaching adulthood, blood vessels are generally latent and angiogenesis is stimulated only in particular conditions such as open wounds, hypoxia and the cycling ovary during pregnancy [1]. This multi-step process is highly regulated via the spatial and temporal distribution of stimulators and inhibitors [1]. Aberrant regulation of angiogenesis has been implicated in more than 70 diseases and the list continues to grow [1]. Insufficient angiogenesis not only impedes healing and regeneration but it also affects the survival of existing cells and tissues. For example, impaired bone healing due to diminished blood vessel formation has been implicated in arthritis, osteoporosis, and non-union fracture healing [2–4], and heart failure can occur from ischemic cardiac tissues [5]. Deficient angiogenesis has also been linked with neurodegenerative diseases. For example, Alzheimer’s disease is characterized by constricted and degenerated vessels, which can cause cerebral infarction and neuronal degeneration [6, 7]. Furthermore, promoting angiogenesis in stroke patients’ brains has been positively correlated with their survival [8] and have been argued as a main target for recovery following ischemic stroke [9].

Given the numerous disorders related to deficient angiogenesis, there is no doubt that therapeutic angiogenesis stimulants will have widespread clinical applications. Current research places a strong emphasis on the delivery of growth factors such as Vascular Endothelial Growth Factor (VEGF), which is currently the most investigated agent for angiogenesis promotion [10]. However, the vasculature induced by VEGF has been reported to be leaky and disorganized [11], emphasizing that in addition to inducing angiogenesis, it is also imperative to stabilize the formed blood vessels. To address these concerns, several approaches, which utilize the combinatorial delivery of VEGF with other growth factors, such as Fibroblast Growth Factor (FGF), Platelet Derived Growth Factor (PDGF-BB), Transforming Growth factor (TGF) and Angiopoietin (Angpt1), have been investigated [12–15]. While growth factors are potent tools to direct and control cell behavior, the classical limitations of protein therapeutics still remain. The short *in vivo* half-life and poor bioavailability at the target site are two main challenges that hamper the successful transition of these protein-based therapeutics from bench to bedside. Although there have been reports of clinical trials involving the protein or gene delivery of VEGF and FGF2, consistent successful outcomes remain elusive and growth factors based therapeutics have not been approved yet for clinical use [10]. The disappointing outcomes from these well-investigated agents urge the need to develop alternative methods to stabilize pro-angiogenic growth factor interactions. Particularly, therapeutic approaches that utilize small molecules to alter cellular function and behavior present an attractive alternative to the pharmacokinetic limitations of protein-based drugs. For example, a sulfated steroid derived from natural products has been reported to promote neovascularization [16] and several other compounds have also been identified to enhance angiogenesis when screened in transgenic zebrafish embryos using a high throughput approach [17]. However, to our knowledge, glycan based approaches have yet to be explored, despite the numerous published studies demonstrating the important role of glycosaminoglycans (GAGs) in cell migration, survival and signaling, all of which are essential processes tightly coordinated during angiogenesis [18].

GAGs play distinctive roles in angiogenesis depending on their type and sequence. They are known to bind to and activate angiogenic growth factors such as VEGF and FGF2 [19]. The binding of VEGF to heparan sulfate (HS) in the extracellular matrix (ECM) guides the extension of tip-cell filopodia and the removal of the HS binding site resulted in deficient vascular branching [20, 21]. GAGs also regulate many aspects of FGF signaling, such as controlling FGF diffusion in the ECM, modulating the interaction between FGF and its receptor at the cell surface, and protecting FGF from proteolytic cleavage [19]. Both chondroitin sulfate (CS) and HS were shown to be important in mediating angiogenesis, and CS and HS have overlapping functions in regulating vessel sprouting [22]. In particular, cell surface heparan sulfate (HS) has been reported to be essential for angiogenesis and its removal inhibits endothelial cell tube formation [23].

The first step in GAG biosynthesis involves the attachment of a xylose sugar to certain serine residues of the core protein component of HS and CS proteoglycans in the late endoplasmic reticulum or cis-Golgi apparatus [24]. Xylosides are small molecules, composed of a xylose sugar and a variable hydrophobic aglycone group at the anomeric carbon, which can act as artificial initiators of GAG biosynthesis without a core protein [25, 26]. Their ability to modulate GAG biosynthesis is well-documented and extensively reported in literature [25–29]. The effects of xylosides are two-fold: Competition with endogenous proteins for the GAG biosynthetic machinery reduces the amount of GAGs, in the form of proteoglycans, anchored on the cell surface or secreted into the ECM while the xyloside-primed GAG chains, secreted out into the ECM, compete with endogenous GAGs outside the cell for binding to various proteins, including growth factors, resulting in varying biological consequences [30–33]. Previously, we have synthesized a class of metabolically stable xylosides using click-chemistry and have shown that these click-xylosides prime GAG chains [30, 31]. The chain length, disaccharide composition, and sulfation pattern of the primed GAG chains have been shown to be dependent on the structure of the aglycone moieties [34]. Moreover, fluoro-xylosides, which are known to inhibit GAG biosynthesis without having any priming activity as they lack a hydroxyl group at the C4 position, block endothelial tube formation [35], further emphasizing the essential contributory role of GAGs. Based on these earlier studies, we hypothesize that the priming of GAG chains by click-xylosides in endothelial cells can have a pro-angiogenic effect.

In this report, we show that click-xylosides not only promote the formation of more extensive endothelial cell networks, but also protect and maintain the network structure in culture. The xylosides were also able to increase blood vessel density in chick chorioallantoic membrane (CAM) assays. Taken together, these results demonstrate that click-xylosides have the potential to be developed as drug candidates for ameliorating various human diseases and medical conditions wherein the promotion of angiogenesis is desired.

## 2 Results

### 2.1 Click xylosides promotes endothelial tube formation on matrigel

**Fig 1:**
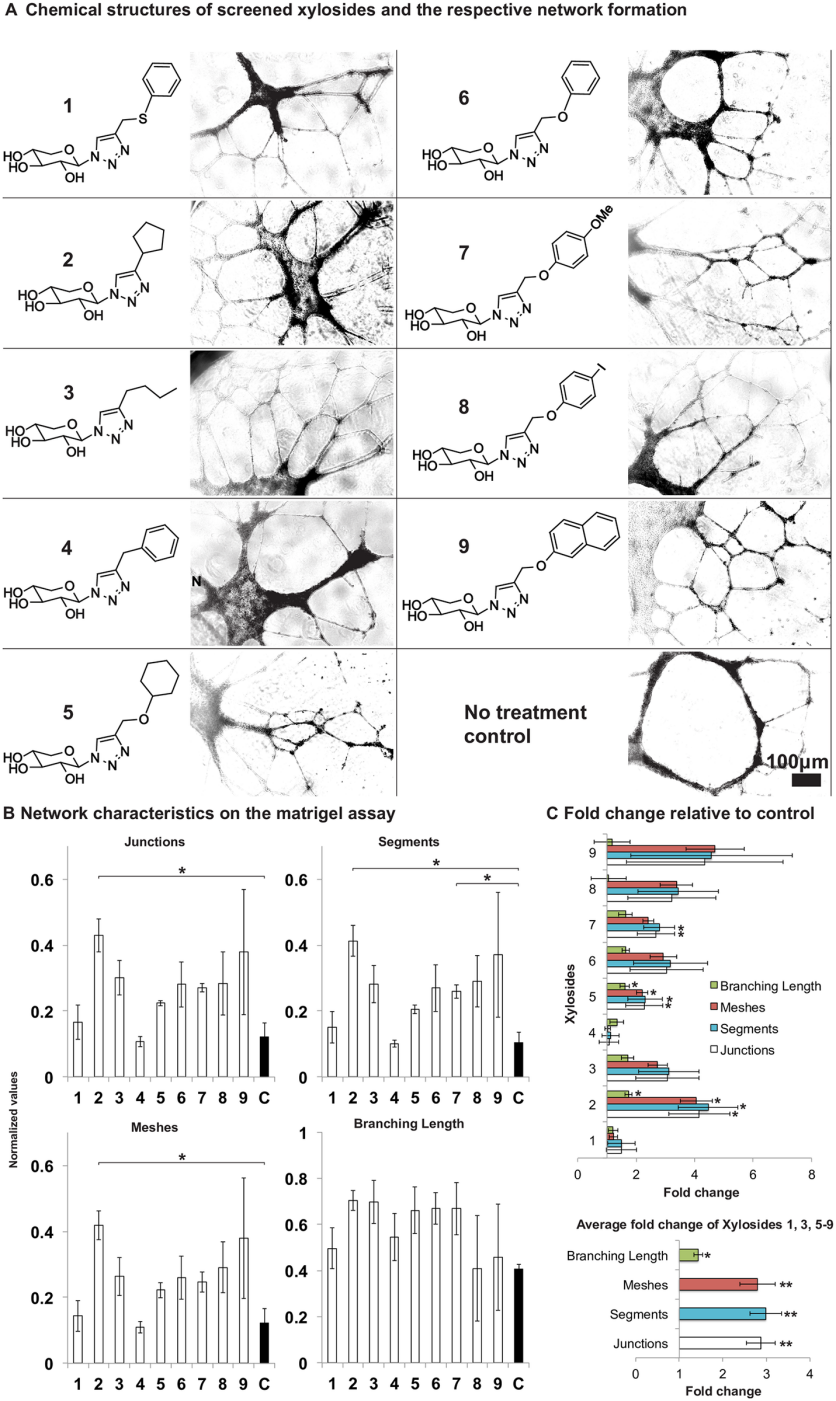
Click-xylosides promoted endothelial tube formation in the matrigel tube formation assay. (A) Nine click-xylosides were preliminary screened for their ability to facilitate HUVEC network formation in matrigel cultures. The respective images were taken at 8 hours after cell seeding. (B) The network properties of the preliminary screening experiment was quantified at 8 hours using the Angiogenesis Analyser ImageJ plugin. The network properties compared are the number of junctions which are branching points, segments which are the tube like elements delimited by two junctions, meshes which are enclosed by segments and the total branching length of the formed network. The data for this preliminary study was averaged over three experiments. No statistical significance were observed between the treatments as determined by one-way ANOVA (S1A Table). Two-sample T-tests (unpaired data with unequal variances) were performed for each xyloside treatment against the untreated control. Statistical significance, as determined by the student T-test against the no treatment control, is indicated by * when p<0.05, ** when p<0.005 and *** when p<0.0005 (S1B Table). (C) TOP: The values were expressed as fold change relative to the no treatment control. Overall, xyloside **2** treatment produced the greatest effect, while xyloside **4** has the lowest fold change. BOTTOM: The other seven xylosides, 1, 3 and 5–7, showed similar effects: the fold changes were averaged over the seven compounds. For the fold change values, a one sample T-test against the control mean value of one was carried out and statistical significance as determined by the T-test is indicated by * when p<0.05, ** when p<0.005 and *** when p<0.0005 (S1C Table). All error bars reflects the standard error.

Nine click-xylosides, each carrying a distinct aglycone group, were screened in matrigel tube formation assays with human umbilical vein endothelial cells (HUVECs) (Fig 1A). These xylosides were chosen for the study based on their high priming activity [34]. Since GAG chains are essential in mediating interactions between growth factors and receptors, the excess secreted GAGs primed by click-xylosides are expected to promote angiogenesis. HUVECs were seeded on matrigel in media containing 100 μM of the tested xylosides, and tube formation was examined after 8 hours. Using the Angiogenesis Analyzer ImageJ plugin [36], we quantified the number of junctions (branching points), segments (tubes delimited by two junctions), meshes (regions enclosed by segments) and total branching length of the cellular networks. For many of the xyloside treated samples, the HUVEC networks were more extensive and well connected in comparison to the untreated controls (Fig 1A). In particular, the most effective promoter, xyloside **2**, had on average, 4.16 times the number of junctions (±1.05), 4.46 times the number of segments (±1.02), 4.06 times the number of meshes (±0.542) and 1.74 times the total branching length (±0.119) as the untreated control (Fig 1B and 1C). On the other hand, the least effective promoter, xyloside **4**, had only 1.07 times the number of junctions (±0.327), 1.12 times the number of segments (±0.293), 1.02 times the number of meshes (±0.092) and 1.34 times the total branching length (±0.240) (Fig 1B and 1C). The remaining seven xylosides have on average, 2.87 times the number of junctions (±0.333), 2.99 times the number of segments (±0.361), 2.79 times the number of meshes (±0.408) and 1.43 times the total branching length (±0.103) (Fig 1C). Based on these results, xylosides **2**, **3** and **4** were tested in subsequent experiments because xylosides **2** and **4** were the most and least effective promoters, respectively, and xyloside **3** was randomly chosen from the remaining moderately effective xylosides.

### 2.2 Investigating the effect of click-xylosides on cell proliferation, viability, migration and morphology

**Fig 2:**
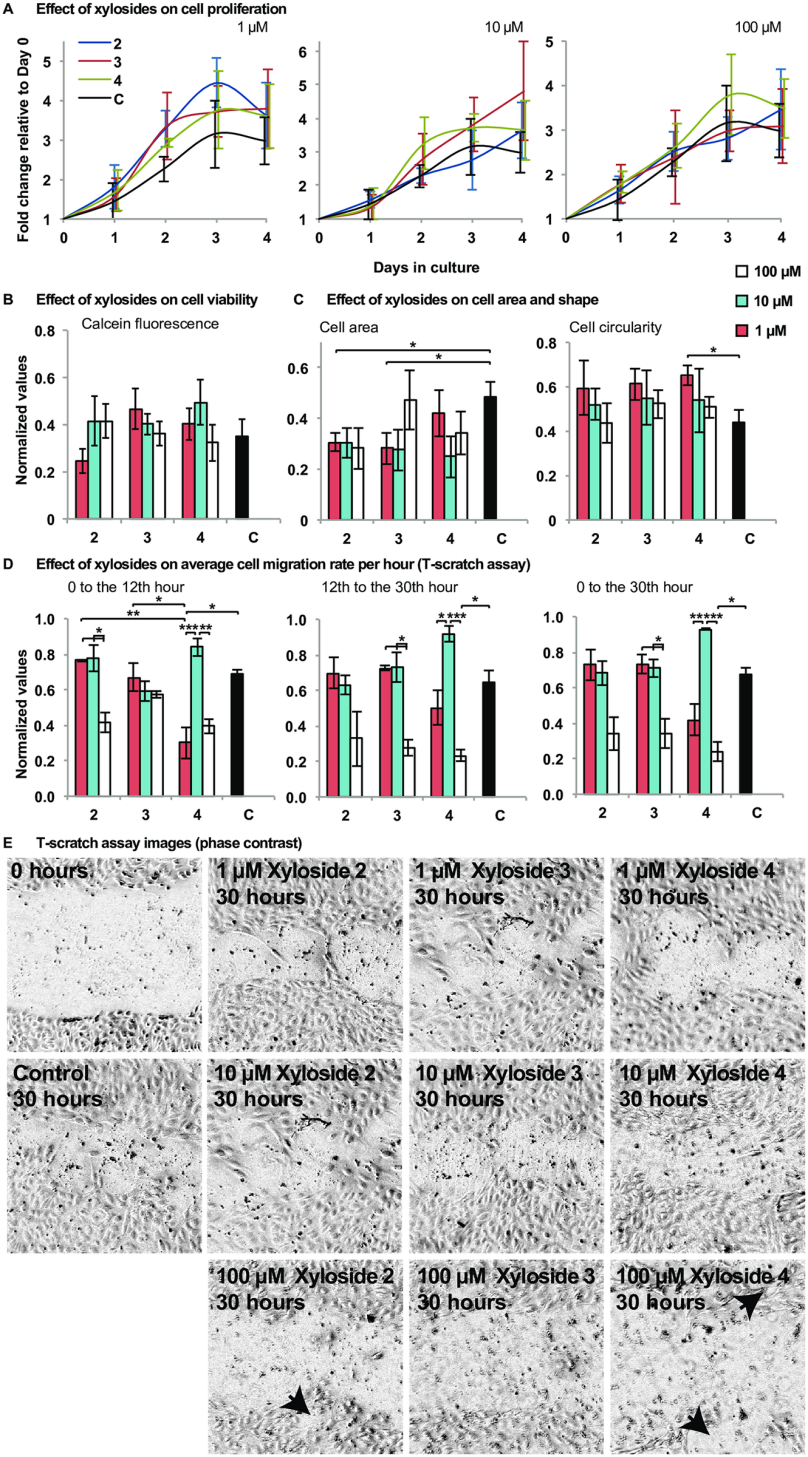
The effect of click-xylosides on cell proliferation, viability, cell migration and cell morpholgy differentially in a concentration dependent manner. (A) Cells were treated with xylosides **2**, **3** and **4** at 1, 10 and 100 μM over a period of 4 days and the number of cells were counted each day. Data shown was averaged over three experiments. Although a small increase in cell number was observed after Day 2 for all xylosides treated at 1 μM, no statistical significance were found with the One-way ANOVA. (B) The cells were labeled with Calcein AM just before treatment with xylosides **2**, **3** and **4** at 1, 10 and 100 μM and after 72 hours of treatment, and the increase in fluorescence were used for comparison. Xyloside treatment did not affect cell viability. Data shown was averaged over six experiments. No statistical significance were found with the One-way ANOVA (S2A Table). (C) The effect of the xylosides on cell migration was determined with the t-scratch assay. Cells were grown to confluence and two scratches were made in each well, forming a cross (t) in the middle. The cells were then treated with the corresponding xylosides. Images of the cross were taken at the 12^th^ and the 30^th^ hour after the scratches were made. Cell area and circularity were obtained from the images of the t-scratch assay at the 12^th^ hour. At least 50 cells were randomly chosen from each image and manually outlined in ImageJ to obtain area and circularity values. Circularity values ranged from 0 (infinitely elongated polygon) to 1 (perfect circle). Data shown was averaged over five experiments. No statistical significance were found with One-way ANOVA (S2A Table). Two-sample T-tests (unpaired data with unequal variances) were performed for each xyloside treatment against the untreated control (S2B and S2C Table). Statistical significance, as determined by the student T-test against the no treatment control, is indicated by * when p<0.05. (D) Migration rates were determined by comparing the area of the cross at the different time points. Data shown was averaged over three experiments. (E) Representative images of the migration assay, 30 hours after scratching and of xyloside exposure. Black arrows indicate the holes left by cell detachment in the 100 μM treatments. One-way ANOVA with post-hoc Tukey’s test was used to determine statistical significance, which is indicated by * when p<0.05, ** when p<0.005 and *** when p<0.0005 (S2D, S2E and S2F Table). All error bars reflect the standard error.

The process of angiogenesis involves the regulation of cell survival, proliferation, differentiation and migration; all of which are processes known to be affected by GAGs. First, to determine if the xyloside treatment affects cell proliferation, HUVECs were incubated with xylosides **2**, **3** or **4** at concentrations of 1 μM, 10 μM and 100 μM, and the number of cells were counted each day. Although a small increase in cell number was observed after 48 hours of xyloside exposure at 1 μM, the xyloside treatment did not significantly affect cell proliferation (Fig 2A). Next a viability assay was conducted after the cells were exposed to the xylosides for 72 hours. The increase in cell number was determined by labeling with Calcein AM and measuring the difference in fluorescence intensity. Cell viability was not affected by the xyloside treatment at all concentrations (Fig 2B).

Next, cell migration was examined using the T-scratch assay. At a concentration of 100 μM, all three xylosides slowed down the rate of migration, and the rate of decrease became more pronounced with time (Fig 2D). The effect was especially significant for xyloside **4**. The rate of migration for the control was 1.49% per hour to the 12^th^ hour (±0.036%), then the rate was reduced to 0.981% from the 12^th^ to the 30^th^ hour (±0.087%). In contrast, the rate of migration when treated with xyloside **4** decreased from 0.963% per hour (±0.070%) to only 0.0352% per hour (±0.28%) (Fig 2D). However, treatment with xyloside **4** at 10 μM increased the migration rate compared to the control, and the difference in rate became more pronounced with time (Fig 2D). Treatment with xyloside **2** and **3** at 1 and 10 μM had similar migration rates compared to the control (Fig 2D).

As notable cell detachment was observed in xyloside treated samples, especially at the highest concentration of 100 μM (Fig 2E), we postulated that the decrease in migration rate is a result of poor cell adhesion to the substrate. Therefore, we examined the morphology of the cells from the migration assay. Cell shape and size are known to differ with different degree of cell adhesion and migration. Cells with less optimal substrate adhesion tend to appear rounder and smaller, while cells that are well adhered show larger surface areas. Actively migrating cells also have elongated morphologies. Although there was minimal statistical significance in the cell morphological data, all xylosides treated cells, with the exception of xyloside **3** dosed at 100 μM, were on average, smaller than the control (Fig 2C). The decrease corresponded to a modest increase in circularity for treatment at 1 μM concentrations of all xylosides (Fig 2C). However, cell circularity seems to decrease with increasing xyloside concentration. Morphological data obtained from the treatments at higher concentration is probably less representative due to notable cell detachment.

In summary, xyloside treatments at lower concentrations, either did not affect or modestly increased migration rates at later time points while treatments at a higher concentration of 100 μM decreased migration rate. In addition, the morphological profiles suggest that when cells are treated with xylosides, they have lower adhesion to the culture plate compared to the control. Taken together, xyloside treatments in a 2-D culture environment affected cell-substrate adhesion and cell migration in a concentration dependent manner, the extent of which will likely differ when examined in a 3-D matrigel culture. Therefore, the effects of the xylosides at different concentrations were further investigated in the matrigel tube formation assay.

### 2.3 The promotion and maintenance of endothelial tube formation by xylosides is dosage dependent and can be attributed to their GAG-priming activities

**Fig 3:**
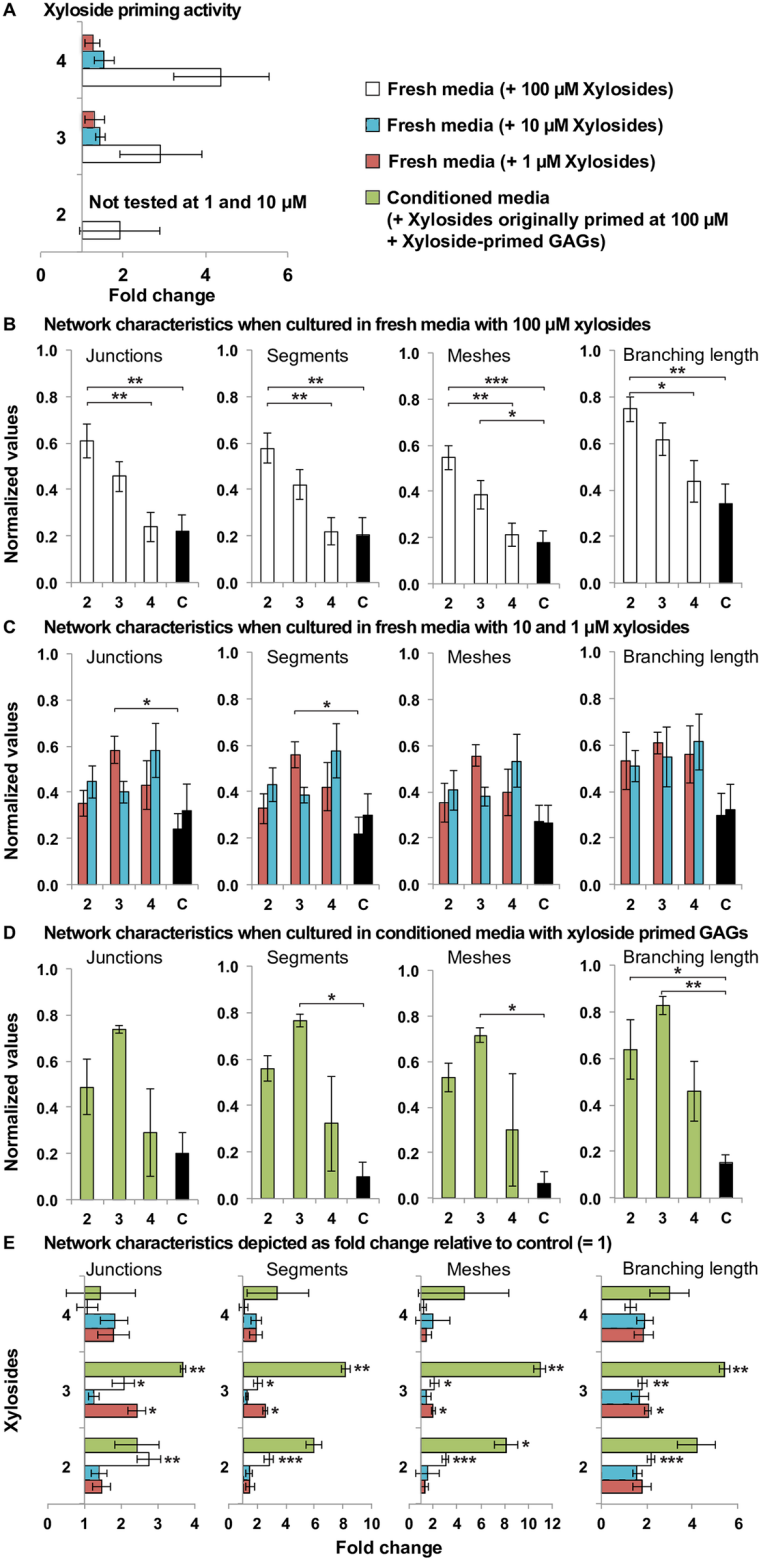
Xylosides and xylosides primed GAGs increases network connectivity of the HUVEC networks on matrigel. (A) Priming activity of the xylosides in HUVECs determined by radiolabeling with [S^35^]-SO_4_ (n = 3) depicted as fold change relative to control (= 1); The network properties of matrigel tube formation assay when cultured in (B) fresh media with xylosides at 100 μM concentration (n = 9), (C) 1 and 10 μM concentration (n = 4 for both concentrations) and (D) conditioned media with unprimed xylosides and xyloside-primed GAGs (n = 3), were quantified after 8 hours with the Angiogenesis Analyzer plugin. Conditioned media was collected from HUVECs treated with 100 μM of xylosides (or no treatment for control samples) for 2 days before use in the matrigel tube formation assay. The network properties compared are the number of junctions that are the branching points, segments that are the tube like elements delimited by 2 junctions, meshes thatare enclosed by segments and the total branching length of the formed network. One-way ANOVA with post-hoc Tukey’s test was used to determine statistical significance (S3B, S3C, S3D and S3F Table). The network characteristics are depicted as fold change relative to the untreated control in (E). One sample T-test against the mean value of 1 (control) was used to determine statistical significance (S3E and S3G Table). Generally, the greatest fold change was seen when the conditioned media was used, indicating that the differences between the treated samples and the untreated control was more pronounced with the conditioned media. The number of experiments conducted is indicated by the stated n value with 3 technical replicates per experiment. Statistically significant difference is indicated by * when p<0.05, ** when p<0.005 and *** when p<0.0005. All error bars reflect the standard error.

**Fig 4:**
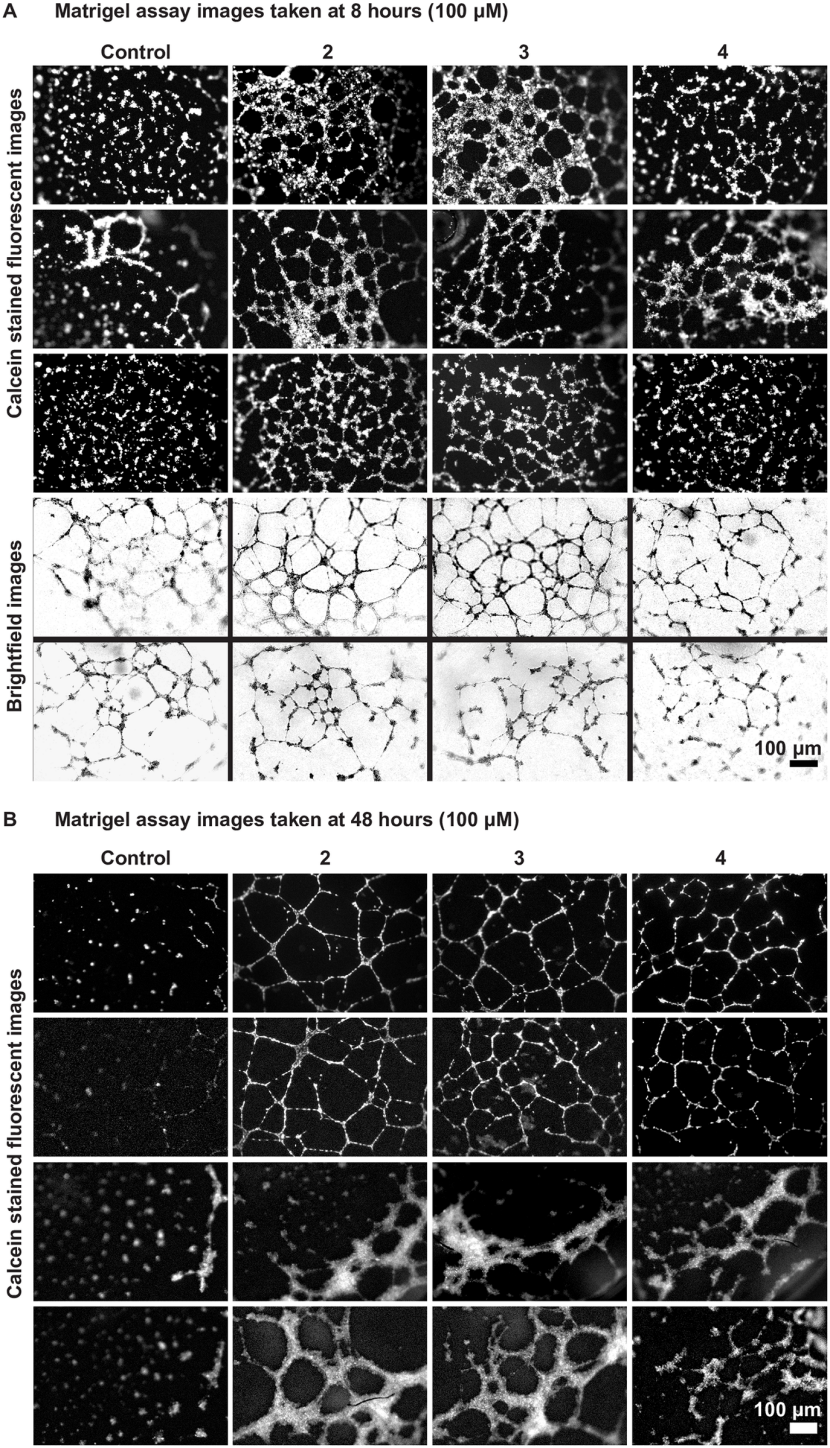
Click-xylosides stabilizes HUVEC networks on matrigel 48 hours after culture. Representative images of calcein stained HUVECs treated with xylosides **2**, **3** or **4** at 100 μM, and a no treatment control on matrigel matrix (A) after 8 hours, and (B) after 48 hours of culture; Images in the same row are from the same experiment.

To understand the effects of the xylosides on endothelial tube formation with respect to dosage and their GAG priming activity, additional experiments were performed on the matrigel culture. First, to affirm the effects and trends observed from the preliminary experiment, the matrigel assay was repeated for the three chosen xylosides at 100 μM and examined at 8 hours and 48 hours after cell seeding. Similar to previous observations, at 8 hours, the HUVEC networks formed by the xyloside **2** and **3** treated cells were more extensive and well-connected compared to the controls, while xyloside **4** was less effective (Figs 3B and 4A). Xyloside **2**, which was the best promoter, had 2.75 times as many junctions (±0.334), 2.81 times as many tubular segments (±0.314), 3.05 times as many meshes (±0.286), and 2.19 times the total branching length (±0.157) compared to the control. In contrast, xyloside **4** had almost the same number of junctions, segments and meshes, and 1.27 times the total branching length (±0.262) compared to the control (Fig 3B).

Matrigel tube formation assays are typically carried out within 24 hours as cell apoptosis becomes more significant after 24 hours, resulting in network disintegration. Here, the assay was followed up to 48 hours [37]. As expected, after 48 hours, the network has mostly collapsed in the control. In contrast, continuous networks of endothelial tubes were still maintained in all the xyloside treated samples, suggesting that the xyloside treatment not only improves network connectivity but also promotes network stability (Fig 4B).

We postulated that the positive effects of xyloside treatment were primarily attributed to the GAG priming activity of the xylosides. To test this, HUVECs were first grown in media containing 100 μM of each xyloside for 2 days, during which, GAG priming will occur. The conditioned media, containing the secreted xyloside primed GAG chains, was then used in the matrigel tube formation assay. The HUVECs on the matrigel were imaged at 8 hours after seeding, and the networks were quantified with Angiogenesis Analyzer. All three xylosides treated conditioned media promoted more extensive and well-connected networks with greater number of segments and branch points (Fig 3D). Interestingly, xyloside **3** instead of xyloside **2** was the best promoter of angiogenesis in this experiment and significantly promoted network branching. Samples treated with xyloside **3** conditioned media had 3.67 times the number of junctions (±0.0747), 8.18 times the number of segments (±0.306), 11.0 times the number of meshes (±0.486) and 5.40 times the total branching length (±0.246) compared to the control (Fig 3D). Xyloside **2**, the best promoter in the previous experiments when the xylosides were added directly to fresh media (Fig 3B), also had positive effects by triggering greater than twice the number of junctions and more than 5 times the number of branches, meshes and total branching length compared to the control (Fig 3D). Although xyloside **4** had limited effect, these samples also showed increase in the quantified network characteristics (Fig 3D). Results when conditioned media containing xyloside primed GAG chains was used were more pronounced than when fresh media containing only xylosides was used, suggesting that the promotion of network connectivity is correlated to the presence of primed GAG chains (Fig 3E).

Since the presence of the primed GAG chains are important for the observed effects, we postulated that the differential effects of the xylosides in promoting tube formation arise from their different GAG priming activities. We examined the priming activity for the three xylosides by culturing the cells in media supplemented with radiolabeled [S^35^]-SO_4,_ isolating the secreted GAGs in the media and determining the relative amount of GAGs primed by the radioactivity. These xylosides were found to prime GAG chains to different extents and similar to previous reports [34], priming increased with concentration (Fig 3A). At 100 μM, all xylosides stimulated at least twice as much GAGs compared to the control (Fig 3A). Xyloside **2**, the best tube formation promoter at 100 μM, generated 1.92 times the amount of GAGs as the control, while xyloside **3** treated samples made 2.92 times (Fig 3A). Xyloside **4**, the least effective tube promoter, primed the most GAGs at 4.39 times (Fig 3A).

As xyloside priming is concentration dependent, we examined the effect on endothelial tube formation for the three xylosides at lower concentrations of 1 and 10 μM. At lower concentrations, all three xylosides remained effective at promoting network connectivity, although the effect was modest (Fig 3C). In contrast to the results generated at the higher concentration, xyloside **3** at 1 μM was the best tube formation promoter, and this treatment generated 2.42 times as many junctions (±0.244), 2.57 times as many tubular segments (±0.261), 2.05 times as many meshes (±0.157) and 2.05 times the total branching length (±0.159) compared to the control. In contrast, xyloside **2**, which also primes the least (Fig 3A), was the least effective at 1 μM and only promoted 1.47 times as many junctions (±0.238), 1.50 times as many tubular segments (±0.303), 1.30 times as many meshes (±0.325) and 1.78 times the total branching length (±0.413) (Fig 3C). Xyloside **4**, which primes the most (Fig 3A), seems to be more effective than xyloside **2** at the lower concentrations although the difference was not statistically significant (Fig 3C). For xyloside **2** and **4** at 1 and 10 μM, promotion of network connectivity appears to slightly increase with concentration, while xyloside **3** treatment had an opposite trend such that the 10 μM dosage was the least effective (Fig 3C).

Taken together, these results suggest that the click-xylosides can act as promoters of angiogenesis *in vitro* by priming GAG chains. However, the positive effects of these compounds on tube formation are not correlated directly or inversely to either concentration nor extent of priming alone. Instead, the results are dependent on a combination of the aglycone structure, priming activity and concentration of the xylosides. The results at different dosages suggest that too much or too little priming does not benefit tube formation and an optimal concentration, which is different for different xylosides, exists. The xylosides also primes GAGs of different disaccharide compositions and sulfation patterns [34] and this may also contribute to the observed effects. That being said, xyloside **3** was consistently a good promoter of network connectivity at various concentrations and also in the conditioned media experiment. Therefore, xyloside **3** was chosen for further analysis against xyloside **4** to study structure and compositions of their primed GAG chains and their effect in the activation of extracellular signal regulated kinase (ERK) signaling pathway.

### 2.4 Click-xylosides, 3 and 4, primed GAGs differently in terms of HS/CS ratio and dissacharide compositions

**Fig 5:**
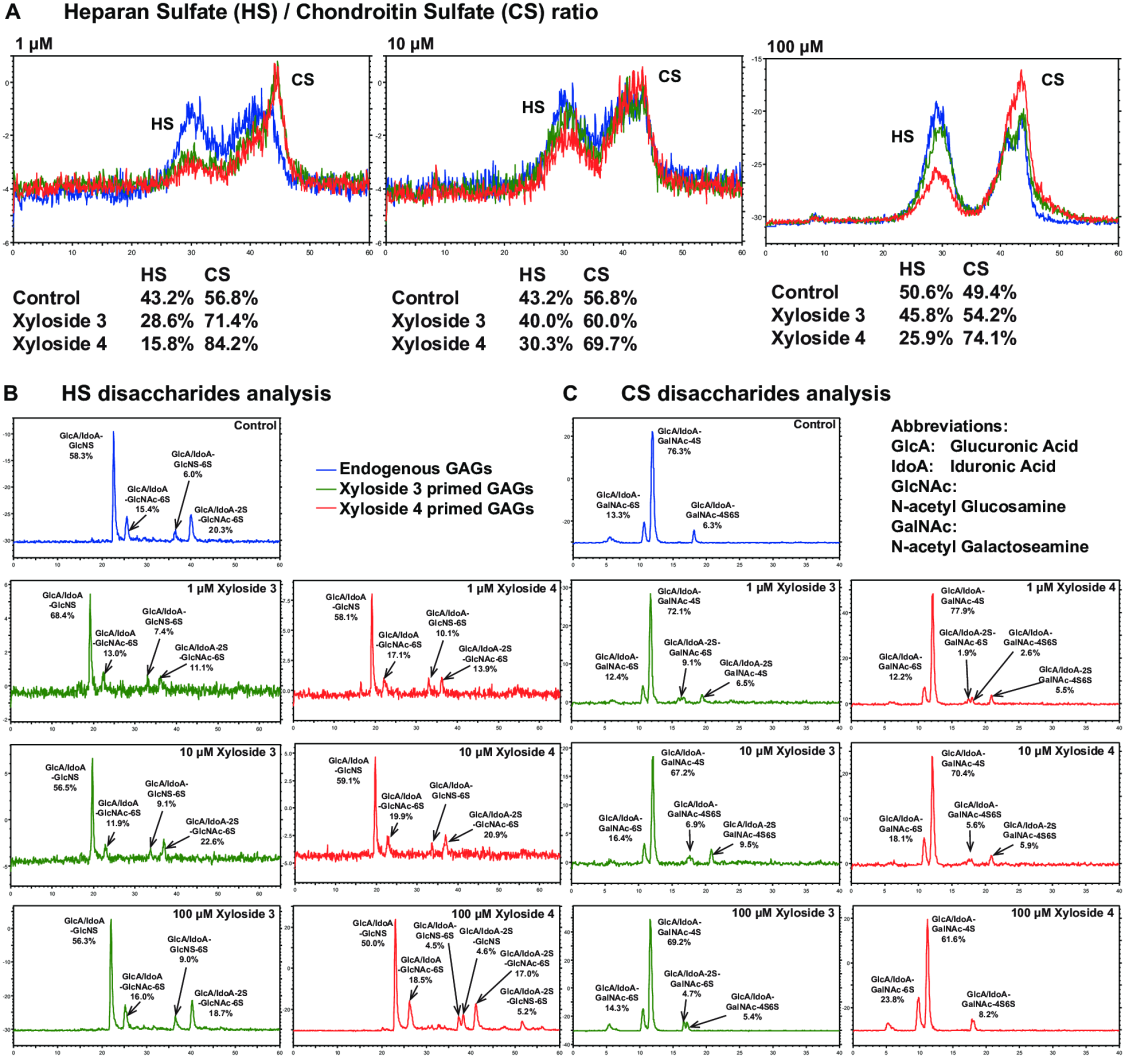
Structural analysis of xyloside primed GAG chains. HUVEC cultures were treated with xylosides **3** or **4** at 1, 10 or 100 μM with [S^35^]-SO_4_ and incubated for 2 days. GAGs from the media and cell extracts were then purified over a DEAE column. BLUE: Endogenous GAGs, GREEN: Xyloside **3** primed GAGs, and RED: Xyloside **4** primed GAGs. (A) 100,000 CPM of purified radiolabeled GAGs when primed at 1 or 10 μM, and 200,000 CPM of GAGs when primed at 100 μM were analyzed separately over a DEAE-3SW column for anion-exchange chromatography. When primed at 10 and 100 μM, xyloside **3** primed GAGs have similar HS/CS ratios to the endogenous GAGs while xyloside **4** primed GAGs have approximately 3–4 times the amount of CS than HS. When primed at 1 μM, both **3** and **4** primed more CS than HS, although **4** still primes relatively more CS than **3** (B). Radiolabeled GAGs were digested with heparitinase I/II/III and analyzed over the CarboPac analytical PA1 column. Generally, xyloside primed GAGs have similar HS compositions to endogenous GAGs with the exception of xyloside **4** primed at 100 μM that has an extra disulfated peak. (C) Radiolabeled GAGs were digested with chondroitinase ABC and analyzed over YMC PA-G column. The CS disaccharides primed by the xylosides have additional disulfated peaks compared to endogenous CS; All peaks were identified by matching with HS and CS disaccharide standards.

Although it has been shown that the presence of the xyloside primed GAG chains is important for the observed results, the effects do not necessarily correlate with the amount of GAG chains primed. We postulated that apart from priming activity, the structure and compositions of the GAG chains primed by the xylosides may be different and are also important contributory factors. GAGs primed from xylosides **3** and **4** were radiolabeled with ^35^S-sulfate and analyzed using High Performance Liquid Chromatography (HPLC). At 10 and 100 μM concentrations, xyloside **3** treated cells was found to produce an almost 1:1 ratio of heparan sulfate (HS) to chondroitin sulfate (CS) which is very similar to the endogenous GAGs (Fig 5A). On the other hand, at all concentrations, xyloside **4** primed GAGs have approximately 3 to 4 times more CS than HS (Fig 5A).

The sulfated GAGs were isolated, digested with heparitinase and chondroitinase enzymes, and analyzed for their disaccharide compositions. The disaccharides composition of the xylosides treated cells differs from the control with the aglycone group. At all concentrations, xyloside **3** primed GAGs with similar HS disaccharide compositions as endogenous HS. On the other hand, an additional disulfated HS peak was identified in the chromatogram of xyloside **4** primed at 100 μM (Fig 5B). In addition, at all concentrations, xyloside **3** primed CS has a similar 4-*O*-sulfation/6-*O*-sulfation ratio to endogenous CS while xyloside **4** primed CS at 10 and 100 μM have lower 4-*O*-sulfation/6-*O*-sulfation ratio than the endogenous CS (Fig 5C). A CS dissacharide peak, disulfated at the 2-*O* and 6-*O* position and not present in the control, was identified in the xyloside **3** primed CS at 1 and 100 μM concentration and in xyloside **4** primed CS at 1 μM. Xyloside **3** primed at 10 μM and xyloside **4** primed at 1 and 10 μM also have an additional trisulfated CS disaccharide peak.

To summarize, HS/CS ratio and disaccharide compositions of the primed GAG chains are dependent on a combination of dosage and the xyloside aglycone group. This likely contributes to the observed differential effects of the xylosides on endothelial tube formation.

### 2.5 Cellular network stabilization by xylosides involves the ERK mediated signaling pathway

**Fig 6:**
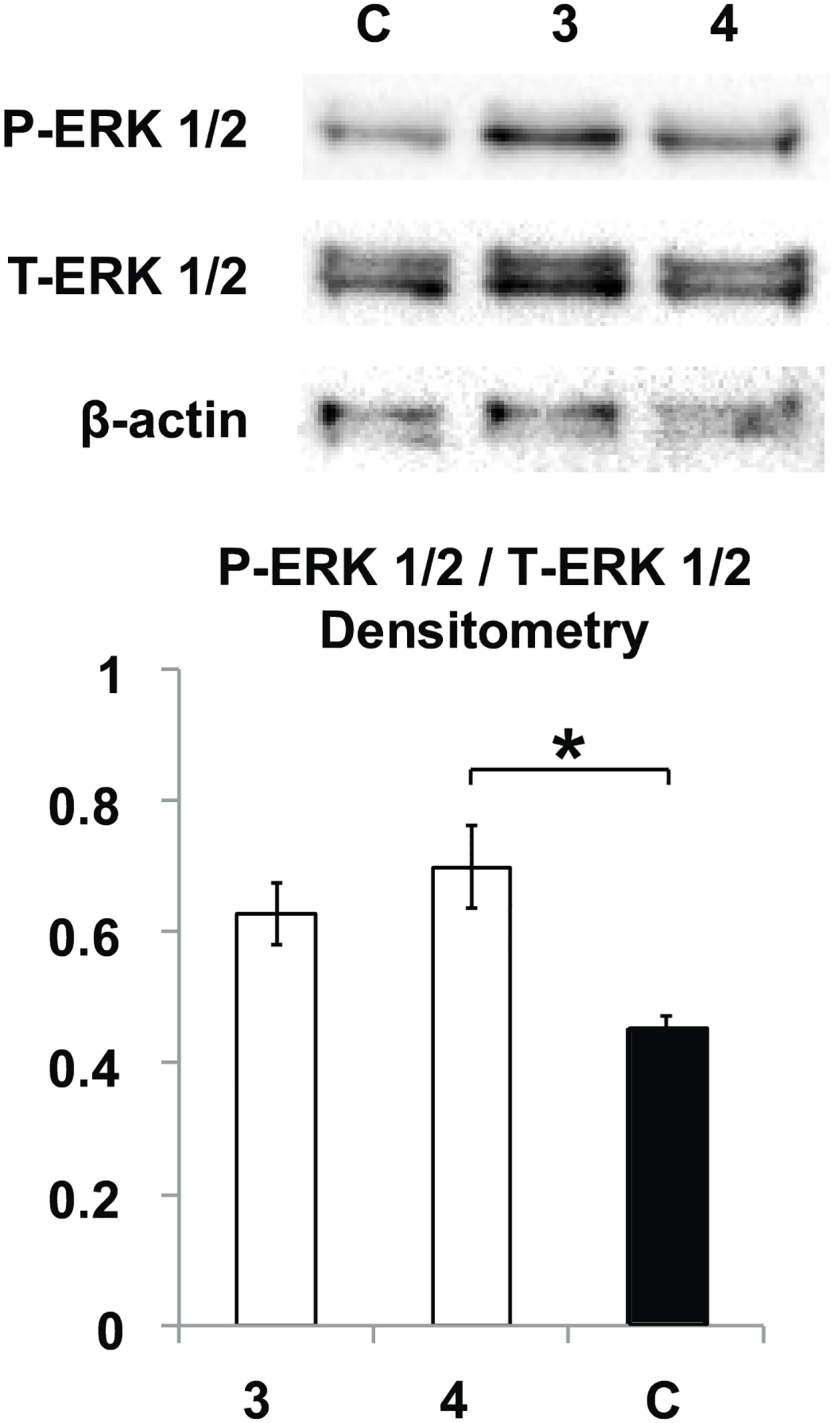
ERK1/2 signaling is increased in xylosides treated HUVECs. Western blot analysis revealed that samples treated with xyloside **3** and **4** at 100 μM for 48 hours have a higher proportion of phosphorylated ERK1/2 compared to the untreated control. Densitometry results are average of 4 experiments. Significant difference is indicated by * where p < 0.05 (S4 Table). Error bars reflect the standard error.

We postulated that the stimulation of angiogenesis seen in the matrigel assays arises from the stabilization of VEGF and FGF2 signaling by the primed GAG chains. VEGF and FGF2 are strong activators of the extracellular signal regulated kinase (ERK) pathway, which has been known to regulate cell migration, proliferation and survival [38]. We investigated the expression levels of phosphorylated ERK1/2 (pERK1/2) on HUVECs incubated with the media containing xylosides **3** or **4** at 100 μM after 48 hours. While both xylosides were able to stabilize the network after 48 hours, xyloside **3** promoted much better network connectivity than xyloside **4**. Therefore, these xylosides were used to study the contribution of the ERK1/2 signaling to the effects observed. Based on the densitometry analysis of the resultant blots, both samples treated with xylosides **3** or **4** had approximately 50% more ERK1/2 phosphorylation than the control (Fig 6). This result suggests that the network stabilization by the xyloside primed GAG chains, in part, acts through the ERK1/2 mediated pathway.

### 2.6 Xyloside 3 loaded collagen gels induce local increase in blood vessel density in chick chorioallantoric membrane (CAM) assays

**Fig 7:**
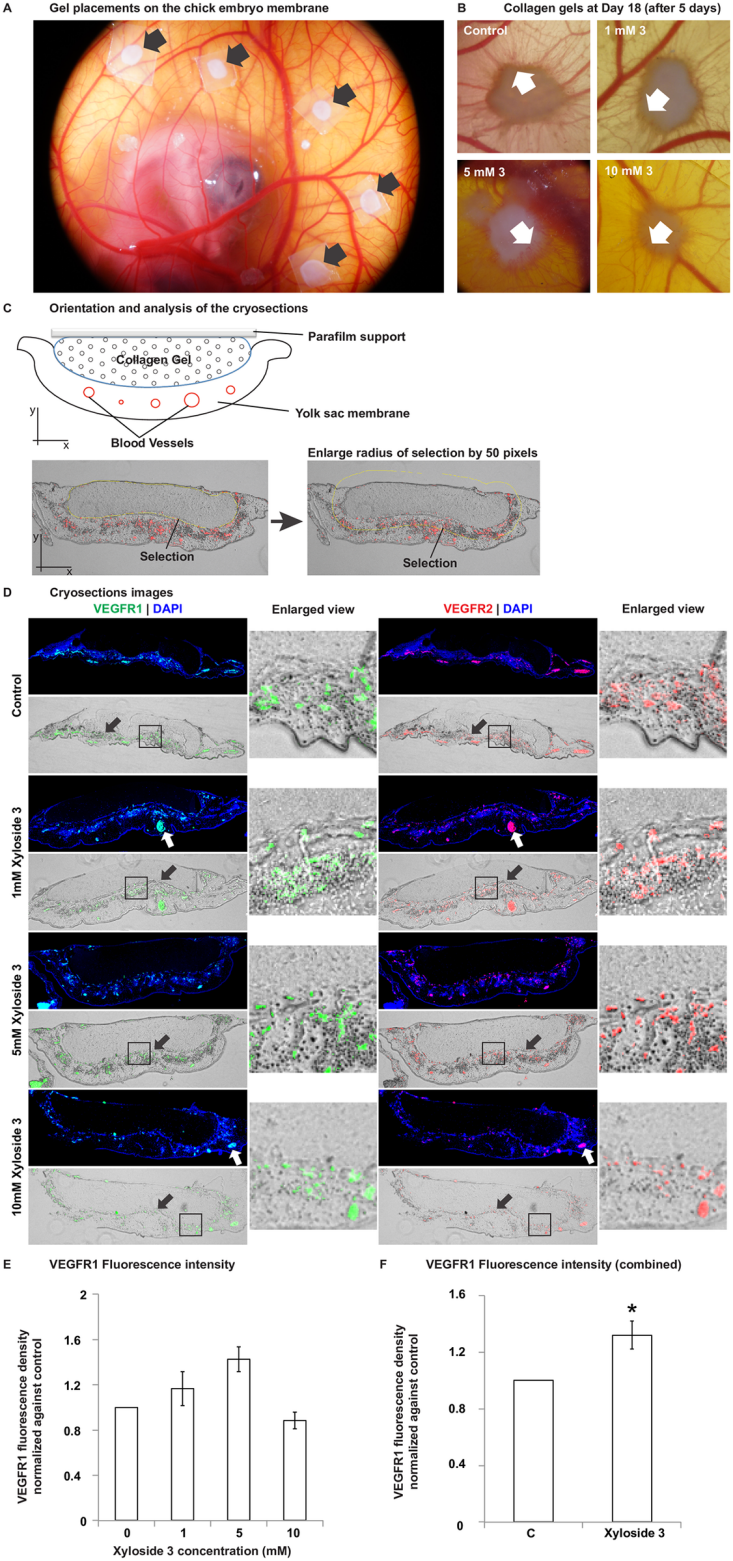
Click-xyloside 3 loaded collagen gels trigger localized increase in endothelial cell density in chick chorioallantoric membrane. (A) Placement of collagen gels on the yolk sac membrane Collagen gels loaded with 1 mM, 5 mM and 10 mM xyloside **3** or DMSO (solvent) control was placed on the yolk sac of the chick embryo at Day 13 of development (Black arrows). Care was taken to ensure that the gels were placed at least 1 cm apart from each other. (B) Ingrowth of blood vessels were observed in the control and the xyloside **3** loaded collagen gels (white arrows). (C) At the end of the experiment, the collagen gels with the chorioallantoric membrane were sectioned in a plane orthorgonal to the topview shown in A and B. 12 μm thick sections were made across the entire gel and at least 20 cross sections distributed evenly across each gel was chosen for analysis of the endothelial cell density around the collagen gel. An area encompassing the gel and a 50 pixels radius border of each image were selected for analysis. The density of endothelial cells was calculated by dividing the total number of red pixels in the selected area (summed over all images of a single gel) by the total number of selected pixels (summed over all images of a single gel). (D) Cross sections of the collagen gels immunofluorescently stained with antibodies against VEGF Receptor 1 (VEGFR1, green) and VEGF Receptor 2 (VEGFR2, red) for endothelial cells, and DAPI (blue) for nuclei, and brightfield images of the same cross sections overlaid with VEGFR1/VEGFR2 staining. White arrows point to pre-existing large blood vessels. Black arrows point to the interface between the collagen gel and the membrane, arrow head points in the direction of the membrane. The enlarged view of the area enclosed by the black box in the brightfield images are also displayed. (E) The density of the VEGFR1 fluorescence around the gel was calculated and normalized to the control gel of each embryo. The normalized values were averaged between 3 samples for the 1 mM and 10 mM. Data shown for 5 mM is over 2 samples. (F) The 1 mM and 5 mM xyloside **3** samples were grouped together and averaged. A one sample T-test were performed with the grouped data against control with a mean of 1 (S5 Table). The treated group has significantly higher endothelial cell density compared to the control group. Significant difference is indicated by * where p < 0.05. Error bars reflect the standard error.

Based on the promising results obtained from the *in vitro* experiments, xyloside **3** was chosen for further studies in chick chorioallantoric membrane (CAM) assays. Xyloside **3** loaded collagen gels of different concentrations and a control gel were placed on the yolk sac of day 13 embryos and incubated for 5 days or less (Fig 7A). From microscopic observations, blood vessels can be seen growing towards and around the collagen gel upon reaching the desired time point (Fig 7B).

At the end of the experiment, the gel was fixed, cryosectioned and served for immunohistochemical staining for endothelial cells using anti-VEGFR1 and anti-VEGFR2 antibodies (Fig 7C and 7D). These cells were positive for both VEGFR1 and VEGFR2, clearly showing that these positive cells are endothelial cells. VEGFR1 was used for quantification due to brighter signal (S1 Fig). At least 20 images evenly distributed across each gel were picked for analysis. For each image, the density of the VEGFR1 staining was quantified in an area that includes the gel and a border with a 50 pixels radius. The values were summed over all the images of each gel and normalized against the control gel of the corresponding embryo (Fig 7E and 7F). The density of endothelial cells around the gel increases with the concentration of xyloside **3** from 1 mM to 5 mM by 20%. However, increasing the concentration of xyloside **3** to 10 mM did not increase the density of endothelial cells around the gel but instead, slightly decreased the density when compared to the control (Fig 7E and 7F).

## 3 Discussion

In the present study, we have shown with both cell culture and *ex ovo* experiments that click-xylosides, which function as primers of glycosaminoglycans (GAGs), promote endothelial tubulogenesis and stabilize the formed network in part by activating the ERK1/2 signaling pathway. Taken together, the results indicated that the xylosides screened here have great potential to be developed as novel therapeutics for promoting angiogenesis.

Xylosides are small molecules in which a distinct aglycone group is attached at the anomeric carbon of a xylose sugar. They can act as artificial initiators of GAG biosynthesis and their effects are known to be two fold. First, xylosides prime GAGs of their own that are secreted into the ECM to influence extracellular interactions and growth factor signaling. Second, xylosides act as decoys by competing with endogenous proteoglycans for GAG biosynthesis enzymes and sugar/sulfate donors, and thereby, reducing the amount of proteoglycan associated GAGs on the cell surface and in the ECM. The latter decoy effect becomes more apparent with higher xyloside concentrations. As GAGs are known to regulate a plethora of cellular processes, the primary goal of this work is to exploit the priming effect of xylosides for promoting angiogenesis. To this end, nine click-xylosides were initially screened at 100 μM concentration in the matrigel tube formation assay. It was found that the majority of the xylosides were able to improve, with varying degrees, the connectivity of the formed networks. From the screening results, xylosides **2**, **3** and **4** were chosen for further indepth analyses. At 100 μM concentration, xyloside **2** and **3** effectively promoted the formation of more extensive and well-connected cellular networks whereas xyloside **4** had limited effect. In addition, while most of the endothelial tubes in the control wells were found to be disintegrated after 48 hours, the endothelial networks in the all the xyloside treated cells were maintained on the matrigel, indicating that the tested xylosides also stabilize the formed tubes.

Angiogenesis involves the coordination and regulation of several processes such as sprouting initiated by the degradation of the surrounding ECM, cell survival, migration and organization into tubular structures. To better understand the contribution of the xylosides in mediating these processes, we examined the effects of xylosides on cell viability, cell proliferation and cell migration. The xyloside treatments did not significantly affect cell viability and proliferation. Conversely, all xylosides slowed down migration when dosed at the highest concentration of 100 μM, and the effect became more pronounced when cells were cultured for longer periods. As the wells treated with 100 μM xylosides had notable cell detachment (Fig 2E), we postulated that the decreased migration resulted from lower cell adhesion to the culture plate. This observation suggested that, the decoy effect of xylosides may be more pronounced at 100 μM leading to the low levels of cell surface GAGs, which are essential for cell-substrate interactions. To probe this possibility, we quantified cell area and circularity as poorly adhered cells usually appears to be smaller and rounder than well-adhered ones. With the cells treated at 1 and 10 μM, we observed an overall modest decrease in cell area and an increase in cell circularity suggesting that xyloside treatment might have perturbed cell-substrate interactions and thereby, reduced cell migration. Although integrin mediated cell-substrate interaction has been shown to be essential for angiogenesis [39], the degree of adhesion differentially affects cell behavior. Particularly, the progressive decrease of cell-matrix adhesion transitions an endothelial cell from a proliferative state (spread) to a tubulogenic state (unspread) which favors angiogenesis, and ultimately to an apoptotic state (round) [40, 41]. It has also been demonstrated that the unspread state results in an VEGF-induced upregulation of genes important for matrix remodeling and vascular invasion [42]. As matrigel comprises many ECM proteins, cell-matrix interactions are expected to be stronger than those in the simple 2D assay on the tissue culture plate. Thus, in the matrigel assays, the decoy effect of xylosides may less likely cause complete cell detachment but instead keep the cells in the unspread state. Taken together, this suggests that the reduction of cell-substrate adhesion due to the decoy effect of xylosides may have led to decreased cell migration in the 2D t-scratch assays whereas this phenomenon may likely contribute to the increased tubulogenesis observed in the matrigel assays.

**Fig 8:**
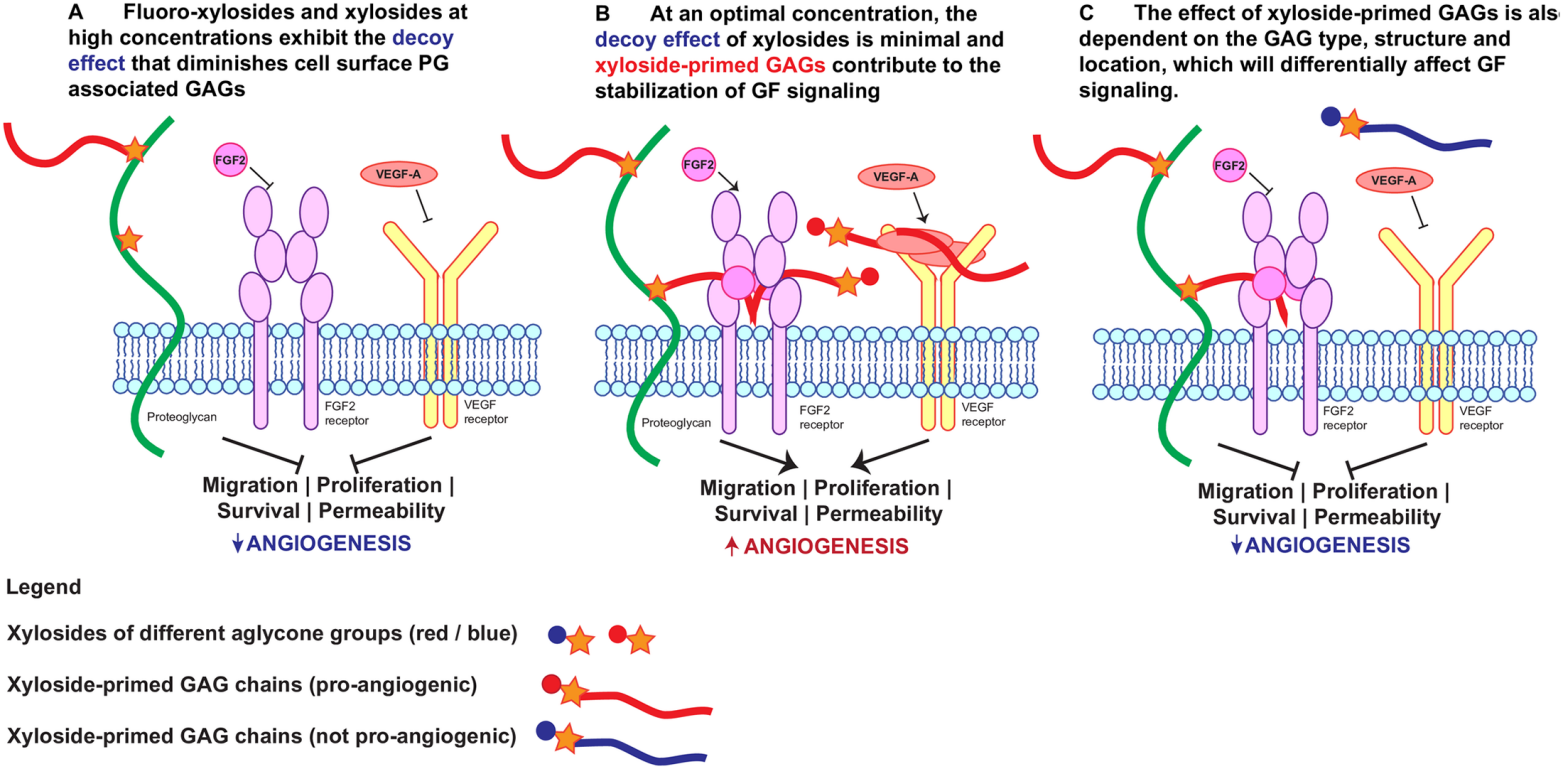
Model of xyloside effects on angiogenesis: When dosed at the optimal concentration, xylosides exhibit minimal decoy effect and prime GAG chains that can contribute to growth factor stabilization and signaling, thereby, promoting angiogenesis. (A) Fluoro-xylosides and xylosides dosed at high concentrations can compete with endogenous proteins for the GAG biosynthetic machinery. This reduces cell surface proteoglycan associated GAGs which are necessary for VEGF and FGF2 signaling. (B) When dosed at the optimal concentration which exploits the GAG priming activities and minimizes the decoy effect, xylosides can promote angiogenesis through the stabilization of VEGF and FGF2 signaling. (C) While the overall increase in the amount of GAG chains is a great contributory factor to promoting angiogenesis, the type and structure of GAGs primed are also important to potentiate growth factor (GF) signaling.

In contrast to a 2D culture system where the primed GAG chains are secreted into the medium, primed GAG chains are likely to have lower diffusivities and stronger interactions with the matrigel matrix components, and remain in the immediate glycocalyx and/or ECM to exert a more pronounced effect on cell behavior. We argue that the promotion of endothelial tube formation on matrigel by xylosides can mainly be attributed to a balance of their GAG priming activity and the decoy effect. As priming activity is known to be concentration dependent, we investigated the effects of xylosides at different dosages on endothelial tube formation. Though all three xylosides remained effective at promoting angiogenesis at the lower concentrations of 1 and 10 μM, some differing trends from the 100 μM treatment emerged. Xyloside **2**, the best promoter at the higher concentration, was the least effective at lower concentrations. Xyloside **4**, which had limited effect at the higher concentration, was more effective than or comparable to xyloside **2** in promoting tube formation at lower concentrations. The high priming activity of xyloside **4** suggests a corresponding large decoy effect and therefore, a consequential reduction in the amount of cell surface GAGs, found in the form of proteoglycans, required for tube formation [23, 35, 43] (Fig 8A). This is congruent with our previous study which showed that the inhibition of GAG biosynthesis by fluoro-xylosides blocks endothelial cell tube formation [35]. Unlike xylosides, fluoro-xylosides have a fluorine instead of a hydroxyl group at the C4 position, and this prevents the molecule from priming GAGs. Therefore, fluoro-xylosides compete with the endogenous proteins for GAG biosynthesis without making any additional GAG chains. These fluoro-xylosides, as one would expect, have been shown to inhibit tube formation in matrigel assays [35]. Conversely, at lower concentrations of xyloside **4**, the decoy effect becomes inconsequential and the primed GAGs aids in branching and tube formation via the activation and stabilization of growth factor signaling (Fig 8B). On the other hand, xyloside **2** that primes the least was less effective at the lower concentrations due to the overall lower amounts of GAGs produced.

To further demonstrate the contributory effects of the primed GAG chains to endothelial tube formation, cells were first incubated with the xylosides at 100 μM for 2 days and the conditioned media, which consists of both unprimed xylosides and xylosides-primed GAGs, was used in the matrigel assay. Compared to the fresh medium containing xylosides, the stimulatory effects of the primed GAG chains containing the conditioned medium on network formation was much more pronounced for all three xylosides, emphasizing the importance of the GAG priming activity in promoting the formation of well-connected and extensive cellular networks. Interestingly, xyloside **3** was a more efficient promoter than xyloside **2** in the conditioned medium experiment while the opposite trend was observed when cells were exposed to xylosides directly added to the fresh medium. As a better GAG primer than xyloside **2**, xyloside **3** is likely to have a stronger decoy effect which negatively affects tube formation in the fresh media experiments. However, it is important to note that xyloside **3** primes more GAG chains and xyloside **3** conditioned media, therefore, will contain more GAG chains than the conditioned media from xyloside **2** treatment. The immediate availability of these GAG chains have significant positive effects on endothelial tube formation. Likewise, xyloside **4**, which has almost no effect on the network in the fresh media experiments, promoted more extensive tube formation in the conditioned media experiment. Taken together, the data demonstrates that the beneficial effects of xylosides on angiogenesis arises from a balance of the GAG priming activity which promotes tube formation and the decoy effect which discourages it (Fig 8A and 8B).

In addition to the GAG priming and decoy activities of the xylosides, the location of the primed GAGs can also differentially modulate their corresponding biological effects [44]. The GAG chains added from the conditioned media are likely to interact with the matrigel growth factors and molecules more evenly, whereas xyloside-primed GAG chains may be less mobile and stay near the cells or in the glycocalyx. It has been shown that cell surface bound proteoglycan-associated GAG chains, ECM bound GAG chains, and free, soluble GAG chains all can differentially affect angiogenesis [44]. Proteoglycans-associated GAGs mostly mediate receptor-growth factor interactions at the cell surface whereas ECM-associated GAGs acts as a reservoir for many growth factors by sequestering and protecting them from proteases. Moreover, GAGs are known to contain sorting information and play important roles in the apical and basolateral sorting of proteoglycans in polarized cells [45]. It has been reported that the xylosides, depending on the cell type and the aglycone group, can preferably increase GAG production on either the apical or the basolateral surface of epithelial cells [46]. Endothelial cells, alike epithelial cells, are polarized with a luminal and abluminal side. Simulations from a mathematical model predict that VEGF binds predominatly to VEGFR1 on the abluminal surface where receptor signaling generally takes place and to VEGFR2 on the luminal surface [47]. The differential signaling that is predicted to occur on the two sides of the polarized endothelial cells indicates that the location where the xylosides-primed GAGs are released can be an influencing factor in determining the results observed. Further investigation would be necessary to elucidate the relationship between xyloside structures and their effects on angiogenesis in terms of the location of the primed GAGs.

The xylosides are known to prime distinct GAG structures depending on their aglycone group and concentration. Earlier reports have suggested that soluble GAGs in the cellular environment, depending on the structure and concentration, can either promote or inhibit angiogenesis and HUVEC proliferation [48, 49]. For example, high concentrations of heparin or HS has been shown to inhibit ocular angiogenesis in young mice, and HUVEC proliferation and tube formation *in vitro* by sequestering VEGF [48, 49]. Lower dosages, depending on the degree and type of sulfation, could either increase or decrease HUVEC proliferation *in vitro* through the mitogenic activity of VEGF [48, 49]. It is likely that the promotion of angiogenesis observed is not simply attributed to the amount of secreted GAGs alone but also to the type of GAGs produced and their sulfation pattern (Fig 8C). Therefore, the structures of the primed GAGs from xylosides **3**, a consistently good promoter, and **4**, a good promoter only at low concentration, were compared along with endogenously produced GAGs from control experiments. Analysis of radiolabeled, primed GAG chains from the control and xyloside treated cells indicated that xyloside **3** produces more heparan sulfate (HS) than chondroitin sulfate (CS) and the ratio of HS/CS ratio is closer to that of endogenous GAGs whereas xyloside **4** consistently produces much more CS than HS, suggesting that a balanced production of HS and CS is important for the beneficial effects observed.

Subtle variations in both HS and CS disaccharide composition of endogenous and xylosides **3/4**-primed GAGs were also observed. The minimal pentasaccharide region that is required for FGF-2 binding consists of *N*-sulfates and a single 2-*O*-sulfate [50]. Although the 6-*O*-sulfate is not necessary for FGF-2 binding, it is essential for potentiating FGF-2 activity [51]. On the other hand, 6-*O*-sulfation and *N*-sulfation have been shown to be sufficient for binding to VEGF and 2-*O*-sulfation is critical for VEGF mitogenic activity [48]. The disaccharide compositional analysis of endogenous GAGs and xylosides-primed GAGs showed the presence of all three sulfate groups necessary for VEGF and FGF2 activity. Xyloside **3**-primed GAGs at all concentrations have very similar HS disaccharide compositions as the endogenous HS. The positive angiogenic effects observed are likely because of the overall increased production of HS that stabilizes and activates both VEGF and FGF2 signaling. Although only a quarter of xyloside **4**-primed GAGs are HS, compared to half in endogenous GAGs, the amount of HS produced still exceeds that of endogenous HS due to the good priming activity. As xyloside **4** promoted tube formation at lower concentrations and in the conditioned media experiments, and have similar HS disaccharide compositions to the xyloside **3** and endogenous HS, we postulated that the primed HS from xyloside **4** is still likely to be pro-angiogenic and the limited beneficial effect at the higher concentration can be mainly attributed to the decoy effect. CS has also been shown to be important for angiogenesis and functionally overlaps with HS [22]. It has been demonstrated that the treatment of endothelial cells with chondroitinase A, B and C led to reduced sprouting in the matrigel tube formation assay [52]. Dermatan sulfate (DS) or CS-B released from proteoglycans following wound healing is important for FGF2-mediated cell proliferation and this activity is mainly linked to DS chains consisting of repeating disaccharide units that comprise of iduronic acid and 4-*O*-sulfated galactosamine [53]. In our analysis of CS disaccharide compositions, both endogenous CS and xylosides-primed GAGs from HUVECs have a major 4-*O*-sulfated peak. We speculated that the increased CS production, in particular the 4-*O*-sulfated disaccharide, due to the priming activity could be partly responsible for the maintenance of the cellular network by both xylosides. Although CS has been shown to largely promote angiogenesis, it has been demonstrated that CS-E, specifically, which consists of both 4-*O* and 6-*O* sulfate groups can compete with HS for binding to VEGF, and this has been suggested to prevent the binding of VEGF to its receptor and thereby, block VEGF signaling [54]. In addition to the decoy effect, the positive effects of the xyloside **4**-primed HS and CS could also be countered by the higher percentage of CS-E that was primed. Although the difference is modest and seems inconsequential, it is magnified by the high priming activity of **4** with a preference for CS. Likewise, a higher percentage of CS-E was also found to be primed by xyloside **3** at 10 μM, which was the least effective concentration for this particular xyloside.

To summarize, the beneficial effects of xylosides on angiogenesis likely arise from the overall increase in pro-angiogenic HS and CS structures that stabilize and activate FGF2 and VEGF interactions with their receptors (Fig 8B and 8C). Both xyloside-primed and endogenous HS chains carry *N*-sulfated, 2-*O*-sulfated and 6-*O*-sulfated disaccharide units that are known to be essential for FGF2 and VEGF activity. Likewise, xylosides-primed and endogenous CS are majorly 4-*O*-sulfated, a structural feature that plays a key role in potentiating FGF2 activity.

The binding of both VEGF and FGF2 is known to stimulate the ERK mediated pathway and has been implicated in the proliferation and migration of endothelial cells [38]. Activation of the ERK1/2 pathway has been shown to result in increased endothelial cell proliferation, migration, differentiation, and to decreased apoptosis [38]. When cells were incubated with xyloside **3** and **4**, increased phosphorylation of ERK1/2 was observed. Although xyloside **3** promoted much better network connectivity than **4**, both xylosides were shown to maintain the network structure *in vitro*. This suggests that the promotion of cell survival and proliferation, and thus the stabilization of the cellular network by the xyloside-primed GAGs involves the activation of the ERK1/2 pathway. This is consistent with the previously mentioned supposition that the overall increased production of 4-*O*-sulfated CS by both xylosides increased FGF2-mediated cell proliferation. In addition, the lack of quantitative correlation between extent of ERK1/2 phosphorylation and improved network connectivity suggests that underlying mechanisms involved are more complex. This is not surprising as other signaling cascades such as the phosphatidylinositol 3-kinase/Akt pathway have also been implicated in angiogenesis and cell survival [38]. As several growth factors are known to have GAG binding sites, it therfore seems likely that the secreted GAG chains influence more than one signaling cascade, and the observed pro-angiogenic effect results from the sum of these interactions. Defining the specific mechanisms and deciphering other signaling pathways involved warrants further investigation.

Finally, the angiogenic potential of xyloside **3**, a consistently good angiogenic promoter, was investigated with the chicken embryo chorioallantoic membrane (CAM) assay. Collagen gels embedded with xyloside **3** promoted localized increase in blood vessel density around the gel. This increase was positively correlated with the xyloside treatment at concentrations of 1 mM and 5 mM. However, at a higher concentration of 10 mM, a moderate decrease, compared to the control gel, was observed instead. As discussed before, it is likely that at high concentrations, the xyloside molecules compete with endogenous proteins for the GAG biosynthetic machinery, depleting the necessary cell surface proteoglycans associated GAGs that mediate angiogenesis and thereby having a counterproductive effect. This experiment suggests the need for encapsulation of xylosides in a polymeric drug delivery system for a localized angiogenic effect and the importance of dosage in achieving the desired effect.

In summary, we have demonstrated that these glycan-based small molecules, termed click-xylosides, can promote angiogenesis through their GAG priming activity. We have shown that depending on the aglycone nature of the molecule, click-xylosides have the ability to induce extensive and stable cellular network formation in the *in vitro* matrigel tube formation assay, and this is in part due to the secreted, primed GAG chains that activate the ERK1/2 signaling pathway. The small size of xylosides, their tunability and permeability through cells make them ideal candidates for the development of small molecular drugs to promote therapeutic angiogenesis. Current studies on angiogenesis promotion focus mostly on the protein and gene delivery of VEGF, which often induces the formation of leaky and disorganized blood vessels [11]. To circumvent this problem, the co-delivery of VEGF and Angpt-1 had been investigated [15, 55]. It has been shown that Angpt-1 decrease blood vessel permeability by increasing GAG content and the depth of the glycocalyx, demonstrating another important role of GAGs in blood vessel formation [56]. Therefore, as oppose to protein and gene delivery methods, xylosides can be much more powerful as therapeutic agents when dosed at the optimal concentration exploiting the GAG priming activity while minimizing the decoy effect (Fig 8). The secreted, primed GAG chains can not only stabilize and activate growth factor signaling but also strengthen the endothelial cell glycocalyx. In addition, these xylosides are also robust tools to study the effect of ECM GAGs on angiogenesis and other biological processes. Future work will focus on the detailed mechanisms through which the xylosides exert pro-angiogenic effects and explore the potential of these GAG biosynthetic modulators for *in vivo* applications.

## 4 Methods

### 4.1 Materials

Xylosides were synthesized as previously reported [30]. All materials are from Sigma Alrich unless specified otherwise. Primary human umbilical vein endothelial cells (HUVECs, Life Technologies), matrigel (Corning), Medium 200 (Life Technologies), low serum growth supplement (LSGS, Life Technologies), Calcein Am, rat tail collagen I (Corning), Alexa-Fluor 488 Goat anti-rabbit secondary antibody and 4',6-diamidino-2-phenylindole (DAPI) were purchased from ThermoFisher Scientific. The primary antibodies used are rabbit anti-Phosphophorylated ERK1/2 from R and D systems, rabbit anti-ERK1/2 from Cell Signaling Technology (CST), mouse anti-β-actin from CST, rabbit anti-VEGF Receptor 1 from Abcam (ab2350), and rat anti-VEGF Receptor 2 from R and D systems (MAB4431). The radiochemical, [^35^S]Na_2_SO_4_, was purchased from MP Biomedicals and flow scintillation mixture, Ultima-FloAP for flow radiometric analysis were obtained from PerkinElmer Life Sciences. DEAE-Sepharose beads were obtained from GE Healthcare. The HPLC columns used were DEAE-3SW column (7.5 mm × 7.5 cm, 10-μm particle size, Tosoh Bioscience LLC), Dionex CarboPac PA1 column (4 × 250 mm, 10-μm particle size, ThermoFisher) and YMC-Pack PA-G column (250 × 4.6 mm, 5-μm particle size, YMC). Protease inhibitor cocktail tablet was purchased from Roche Diagnostics. The chicken eggs used were of the strain Rhode Island Red and purchased from AA Lab Eggs, INC. Experiments were conducted using chick embryos, at stages before hatching, and were performed in compliance with the University of Utah Institutional Animal Care and Use Committee (IACUC) requirements.

### 4.2 Maintenance of human umbilical vein endothelial cells

HUVECs were expanded on T75 flasks in a humidified incubator at 37°C with 5% CO_2_. The maintenance medium is composed of Medium 200 supplemented with LSGS. HUVECs of passage number 2–6 were used for all experiments.

### 4.3 Analysis of xyloside mediated GAG priming activity in HUVECs

HUVECs were seeded at ~100,000 cells per well onto 6-well plates in EGM-2 (Lonza). Upon reaching 50% confluence, the media was replaced with 100 μM of the tested xylosides in EGM-2 with [^35^S]Na_2_SO_4_. 0.1 mCi of radioactivity was used per well. After 48 hours, the media of each well was collected and diluted 3X with wash buffer (0.1 M NaCl, 0.02 M NaOAc, pH 6.8) and loaded over 0.5 ml of DEAE-Sepharose column pre-equilibrated with 5 ml of wash buffer. The columns were each washed with 15 ml of wash buffer and eluted with 5 ml of elution buffer (1M NaCl, 0.02 M NaOAc, pH 6.0). The elution was concentrated and desalted 6 times with 0.5 ml centrifugal filters (3000 MWCO, Amicon, Millipore) to a final volume of 100 μl. The amount of GAG chains primed by various click-xylosides was determined by quantifying the [S^35^] radioactivity incorporated in the purified GAG solution. 5 μl of each sample was diluted with 5 ml of scintillation mixture and measured with a liquid scintillation counter.

Based on the results from the scintillation counter, 200,000 CPM of each sample primed at 100 μM, and 100,000 CPM of each sample primed at 1 and 10 μM was analyzed with high-pressure liquid chromatography (HPLC) coupled to an inline flow scintillation analyzer, over a DEAE-3SW column, for anion-exchange chromatography. For the analysis of disaccharide composition, samples were digested with either a Heparitinase I/II/III or Chondroitinase ABC. Digested heparan sulfate (HS) was analyzed over Dionex CarboPac PA1 column while the digested chondroitin sulfate (CS) was analyzed over YMC-Pack PA-G column. CS and HS disaccharide standards were co-injected to deduce the identity of each peak.

### 4.4 Matrigel angiogenesis assay

Matrigel matrix was thawed on ice at 4°C overnight. 50 μl of Matrigel were added to each well of a 96 well plate and incubated for at least half an hour at 37°C. Cells were trypsinized 48 hours prior to the matrigel angiogenesis assay and seeded on 6-well plates at 50% confluence. The medium was changed to EGM-2 with 100 μM of the tested xyloside. 1 μl of Calcein AM (1 mg/ml) were added to each well of the 6-well plates and incubated for at least half an hour. The HUVECs were then trypsinized and seeded on the Matrigel coated 96 well plate at the density of ~20,000 to 30,000 cells per well. The media used for the assay was either fresh EGM-2 with 100 μM of the tested xyloside or conditioned media which consists of xyloside containing EGM-2 that has been incubated with the HUVECs for at least 48 hours in the 6-well plate.

### 4.5 Image analysis of matrigel assay

At the required time point, one image of each well in the Matrigel coated 96-well plate was captured with a 4 × objective lens on a Olympus IX73 fluorescence microscope. Images were processed with Angiogenesis Analyzer (Gilles Carpentier) in ImageJ (National Institute of Health, Bethesda, MD, USA). For analysis of the formed networks, values returned for the number of segments, junctions, meshes and total branching length were compared and normalized to a 0 to 1 scale dependent on the range of values for each experiment. Junctions refer to branching points, segments are elements delimited by 2 junctions and meshes are areas enclosed by segments forming loops. The normalized values were averaged over at least 3 experiments with 3 replicas in each experiment.

### 4.6 Proliferation, viability and migration assays

To determine if the xylosides affect cell proliferation, HUVECs were seeded onto 6 well plates in EGM-2 medium at a density of 40,000 cells per well. The following day after cell attachment, the cells in 10 of the wells were trypsinize and counted separately using a hemocytometer. The average cell count obtained was assigned as the Day 0 value. The medium in the remaining wells were changed to EGM-2 with 100 μM, 10 μM or 1 μM of the tested xylosides and the cells were returned to the incubator. After 24 hours, one well of each tested condition was trypsinized and counted using a hemocytometer. The resultant cell count was divided over the assigned Day 0 value to get the fold change. Cell counting was repeated for the cells cultured in the tested conditions for 2 days, 3 days and 4 days. The experiment was repeated three times to get an average fold change value for each day in each treatment.

To determine if the xylosides affect cell viability, HUVECs were seeded onto 12 well plates in EGM-2 medium at a density of 50,000 cells per well. After 24 hours following cell attachment, 0.5 μl of Calcein AM (1 mg/ml) was added to each well and incubated for at least half an hour. The cells were then washed once with 1 × Phosphate Buffered Saline (PBS) and fresh medium was replaced. The fluorescence intensity was recorded with a microplate reader (Synergy H1, biotek) with the excitation/emission wavelengths of 485/528 nm. The medium was subsequently changed to EGM-2 with 100 μM, 10 μM or 1 μM of the tested xylosides and returned to the incubator for 72 hours. After 72 hours, the cells were incubated with Calcein AM again for at least half an hour, washed once with 1 × PBS and replaced with fresh medium. The fluorescence intensity was recorded with the microplate reader. The experiment was repeated 3 times in duplicates. For the analysis of cell proliferation rate, the fluorescence intensity at 72 hours was divided by that at 0 hours (before the addition of xylosides). For each experiment, the values were normalized to 0 to 1 scale. The reported mean and standard error is the average of the normalized values.

To determine if the xylosides affect cell migration, HUVECs were seeded onto 12 well plates in EGM-2 medium at a density of 100,000 cells per well. When the wells reached ~90% confluence and the HUVECs formed a monolayer, the medium was replaced with serum free EGM-2 and incubated for 24 hours. Using a 10–100 μL pipette tip, a horizontal and a vertical scratch were made, forming a “t-shaped” gap in each well. The wells were then washed once with 1 × PBS and the medium were replaced with EGM-2 with 100 μM, 10 μM or 1 μM of the tested xylosides. One image of each well with the “t-shaped” gap positioned in the center was captured with a 4 × objective lens on a Olympus IX73 fluorescence microscope. Images were captured again at the same position after 12 hours and after 30 hours of incubation in the xyloside media. The area of the gap was analyzed and measured with ImageJ. The percentages reported are of the gap area at 0 hours.

### 4.7 Analysis of ERK signaling

HUVECs were seeded onto 6 well plates in EGM-2 medium and grown to 50% confluence. The medium was changed to EGM-2 with 100 μM of the tested xylosides. After 48 hours, the cells were placed on ice and washed twice with ice cold 1 × PBS. Then, the cells were lysed and scraped off the plate with 300 μl per well of RIPA buffer (150 mM NaCl, 1% triton X-100, 0.5% sodium deoxycholate, 0.1% sodium dodecyl sulphate (SDS), 50 mM Tris, pH 8.0) supplemented with protease inhibitor cocktail tablet just before use. The scraped cells were incubated in the RIPA buffer at 4°C for 30 minutes and centrifuged at 15000 × g for 30 minutes. The protein samples were dried with a speed vac overnight and resuspended in 20 μl of deionized water. The protein concentration was determined with bicinchoninic acid (BCA) assay kit (Pierce). 30 μg of total protein from each sample was mixed with SDS loading buffer and ran on a 12% SDS polyacrylamide gel at 200 V for 60 minutes. The ran protein samples were then transferred onto Immobilon-P^SQ^ PVDF membranes (Millipore), blocked with 3% bovine serum albumin (BSA) and probed with the primary antibodies. The blot was washed, counter stained with Horse Radish Peroxidase (HRP) linked secondary antibodies, developed with SuperSignal West Pico chemiluminescent substrate (Pierce) and imaged with Biorad Chemidoc imaging system. The band density was quantified with ImageJ (National Institute of Health, Bethesda, MD, USA). The primary antibodies used are rabbit anti-Phosphophorylated ERK1/2, rabbit anti-ERK1/2 and mouse anti-β-actin.

### 4.8 Chicken embryo chorioallantoic membrane assay

Collagen gel solutions (2.8 mg/ml rat tail collagen I in 1 × PBS, 0.02 M Hepes buffer, 0.006 N NaOH) containing 1 mM, 5 mM and 10 mM xyloside **3** or DMSO solvent controls were pipetted into 30 μl droplets in a parafilm lined petri dish and crosslinked at 37°C for 2 hours.

Fertilized Rhode Island Red chickens eggs were acquired from a AA Lab Eggs, INC. (Westminster, California) and incubated in a humidified chamber set at 38°C and 70% humidity. After 2 days of incubation in a horizontal position, a crack was made in the bottom of the shell and the egg contents emptied into a weighing dish. The *ex ovo* cultures were further incubated in a humidified incubator at 38°C for another 11 days. At Day 13, the xylosides loaded and control collagen gels were placed on the yolk sac membrane. Each embryo has one collagen gel of each concentration and one control gel. Care was taken so that no gels were placed directly on major blood vessels and the gels were placed at least 1 cm away from each other (Fig 7A). The embryos were incubated with the gels for 5 days or until the embryo dies.

Upon reaching the desired time points, the gels were cut out from the membrane, fixed with 4% Paraformaldehyde (PFA) for 30 minutes at room temperature and kept at 4°C in 1XPBS. For cryosections preparation, the gels were equilibrated sequentially in 10% sucrose, 30% sucrose and Tissue-Tek O.C.T. Compound. Then, the gels were embedded in the O.C.T compound on dry ice and cut into 12 μm sections with a cryostat. The cuts were made in a plane orthogonal to the plane of the yolk sac membrane (Fig 7C). The sections were left to dry overnight.

For visualization, the sections were washed twice with 1XPBS, blocked with 4% skim milk for 1 hour at room temperature and incubated with rabbit anti-VEGF Receptor 1 antibodies at 4°C overnight. The sections were then washed and incubated with Alexa-Fluor 488 Goat anti-rabbit secondary antibody and DAPI. Subsequently, the sections were incubated with rat anti-VEGF Receptor 2 antibody at 4°C overnight. The sections were then washed and incubated with Alexa-Fluor 594 Goat anti-rat secondary antibody. The latter staining was to ensure that the VEGFR1 positive cells overlapped with VEGFR2 positive cells, demonstrating that they are endothelial cells (S1 Fig).

### 4.9 Image analysis of CAM assay

The sections were captured with a 4 × objective lens on a Olympus IX73 fluorescence microscope and analyzed with ImageJ. At least 20 cross-sections evenly distributed across each gel were chosen for quantification. Using the free hand selection tool, an area emcompassing the gel in each image was selected and its radius enlarged by 50 pixels (Fig 7C). Therefore, the density of the VEGFR1 immunostaining was quantified in an area which includes the gel and a border of 50 pixel radius. The pixel density stained by VEGFR1 was then summed over all the images of one gel and divided against that of the control gel from the same embryo.

### 4.10 Statistical analysis

All data are presented as mean ± standard error (SEM) of at least 3 separate experiments (with triplicates each) unless otherwise specified. One-way ANOVA and Tukey’s post test analysis were performed at confidence interval levels of 95% with KaleidaGraph (Synergy) (S1, S2, S3, S4 and S5 Tables).

## Supporting information

S1 Table Statistical analysis of the data presented in Fig 1. Preliminary matrigel assay experiments with the library of click-xylosides.
(PDF)

S2 Table Statistical analysis of the data presented in Fig 2. Viability assay, cell area, cell circularity, and cell migration of cells treated with xylosides 2, 3, and 4 at 1, 10, and 100 μM concentrations.
(PDF)

S3 Table Statistical analysis of the data presented in Fig 3. Priming activity of xyloside 2, 3, and 4, and characteristics of the formed networks from the *in vitro* matrigel assays.
(PDF)

S4 Table Statistical analysis of the data presented in Fig 6. P-ERK1/2 / ERK1/2 expression of cells treated with xyloside 3 and 4 at 100 μM, and the untreated control (western blot densitometry data).
(PDF)

S5 Table Statistical analysis of the data presented in Fig 7. Quantified VEGFR1 fluorescence from the cryosections of the xyloside 3 loaded collagen gels, placed on the chick chorioallantoic membrane.
(PDF)

S1 Fig VEGFR1 positive cells coincide with VEGFR2 positive cells. Cross sections of the collagen gel on the chick chorioallantoic membrane were immunofluorescently stained with antibodies against VEGFR1 (Green) and VEGFR2 (Red), and DAPI (Blue) for nuclear staining. The staining appears yellow where VEGFR1 and VEGFR2 staining coincides. The arrows indicate lumen formation.
(PDF)
