# Supplementary material for: A glycan-based approach to therapeutic angiogenesis
Source: PLoS One. 2017 Aug 1;12(8):e0182301. doi: 10.1371/journal.pone.0182301 (PMC5538652; doi:10.1371/journal.pone.0182301)
Supplement: S1 Table — Preliminary matrigel assay experiments with the library of click-xylosides. (PDF) [file pone.0182301.s001.pdf]

S1 Table. Statistical analysis of the data presented in Fig 1; Preliminary matrigel assay experiments with the library of click-xylosides.

S1A Table. One-way ANOVA comparing the formed network characteristics formed from cells treated with xylosides 1-9 and the untreated control.

Junctions	$F(9,20)=1.8016$, $p=0.1308$
Segments	$F(9,20)=1.9480$, $p=0.1028$
Meshes	$F(9,20)=1.9070$, $p=0.1100$
Branching length	$F(9,20)=0.9082$, $p=0.5371$

n=3

No post-hoc test was performed, as data was not statistically significant.

S1B Table. Two sample student t-tests comparing the formed network characteristics formed from cells treated with xylosides 1-9 conducted against the no treatment control.

Xyloside	Junctions	Segments	Meshes	Branching length
**1**	t(3)=-0.6688 p=0.5417	t(3)=-0.8202 p=0.4660	t(3)=-0.3382 p=0.7523	t(2)=-0.9935 p=0.4174
**2**	t(3)=-4.6621 p=0.01024*	t(3)=-5.5017 p=0.008271*	t(3)=-4.7511 p=0.008973*	t(2)=-0.9935 p=0.4174
**3**	t(3)=-2.6433 p=0.06005	t(3)=-2.7721 p=0.06734	t(3)=-1.9373 p=0.1295	t(2)=-3.043 p=0.08432
**4**	t(2)=0.3073 p=0.7821	t(2)=0.07796 p=0.9437	t(2)=0.2578 p=0.8154	t(2)=-1.334 p=0.3065
**5**	t(2)=-2.370 p=0.1329	t(2)=-3.0388 p=0.06471	t(2)=-1.9939 p=0.1408	t(2)=-2.4848 p=0.1225
**6**	t(3)=-1.9729 p=0.1335	t(2)=-2.1243 p=0.1336	t(3)=-1.7401 p=0.1662	t(2)=-3.7076 p=0.05228
**7**	t(2)=-3.363 p=0.06117	t(3)=-4.2316 p=0.01791*	t(3)=-2.2901 p=0.0919	t(2)=-2.2845 p=0.1428
**8**	t(2)=-1.5445 p=0.2277	t(2)=-2.2266 p=0.1260	t(3)=-1.8799 p=0.1516	t(2)=-0.01130 p=0.992
**9**	t(2)=-1.3214 p=0.307	t(2)=-1.3864 p=0.2943	t(2)=-1.3619 p=0.2943	t(2)=-0.2228 p=0.8441

n=3

* Statistically significant where $p<0.05$

S1C Table. Fold change of the formed network characteristics formed from cells treated with xylosides 1-9 relative to the no treatment control was tested with single group t-test against the mean value of 1.

Xyloside	Junctions	Segments	Meshes	Branching length
**1**	t(2)=0.86059 p=0.4802	t(2)=0.97006 p=0.4343	t(2)=0.46574 p=0.6872	t(2)=1.0166 p=0.4163
**2**	t(2)=6.0991 p=0.02584*	t(2)=6.553 p=0.0225*	t(2)=6.7909 p=0.021*	t(2)=6.7943 p=0.02098*
**3**	t(2)=3.3955 p=0.07687	t(2)=3.1535 p=0.08755	t(2)=2.4427 p=0.1346	t(2)=3.1063 p=0.08988
**4**	t(2)=-0.88433 p=0.4698	t(2)=-0.23153 p=0.8384	t(2)=-0.69515 p=0.5589	t(2)=1.3569 p=0.3077
**5**	t(2)=12.75 p=0.006096*	t(2)=7.8765 p=0.01574*	t(2)=4.3773 p=0.04843*	t(2)=2.5294 p=0.1272
**6**	t(2)=2.3237 p=0.1458	t(2)=2.3088 p=0.1473	t(2)=1.12169 p=0.1695	t(2)=3.8513 p=0.06129
**7**	t(2)=11.346 p=0.007679*	t(2)=7.6496 p=0.01666*	t(2)=4.0121 p=0.05687	t(2)=2.3171 p=0.1464
**8**	t(2)=1.6907 p=0.233	t(2)=2.3916 p=0.1392	t(2)=2.1644 p=0.1629	t(2)=0.011342 p=0.992
**9**	t(2)=1.3542 p=0.3084	t(2)=1.4042 p=0.2954	t(2)=1.4014 p=0.2961	t(2)=0.22358 p=0.8438
**1, 3, 5-9 (Average)**	t(6)=6.0812 p=0.0008987**	t(6)=6.0086 p=0.0009573**	t(6)=5.0591 p=0.002313**	t(6)=3.8088 p=0.008876*

n=3

* Statistically significant where p<0.05

** Statistically significant where p<0.005
