# Supplementary material for: A glycan-based approach to therapeutic angiogenesis
Source: PLoS One. 2017 Aug 1;12(8):e0182301. doi: 10.1371/journal.pone.0182301 (PMC5538652; doi:10.1371/journal.pone.0182301)
Supplement: S2 Table — Viability assay, cell area, cell circularity, and cell migration of cells treated with xylosides 2, 3, and 4 at 1, 10, and 100 μM concentrations. (PDF) [file pone.0182301.s002.pdf]

S2 Table. Statistical analysis of the data presented in Fig 2; Viability assay, cell area, cell circularity, and cell migration of cells treated with xylosides 2, 3, and 4 at 1, 10 and 100 μM concentrations.

S2A Table. One-way ANOVA of viability assay, cell area, cell circularity experiments.

Viability assay (n=6)	F(9,50)= 0.90442676, p=0.52875
Cell area (n=5)	F(9,49)=1.206, p=0.31856
Cell circularity (n=5)	F(9,49)=0.60214, p=0.7875

No post-hoc test was performed as data was not statistically significant.

S2B Table. Two sample student t-tests were conducted to compare the cell area of xyloside treated cells to the no treatment control cells.

Concentration / Xyloside	1 μM	10 μM	100 μM
**2**	t(6)=-2.6395 p=0.03387*	t(7)=-2.2192 p=0.05728	t(7)=-2.0652 p=0.0767
**3**	t(7)=-2.3951 p=0.04377*	t(7)=-2.1144 p=0.07139	t(7)=-0.086375 p=0.9341
**4**	t(6)=-0.58301 p=0.5792	t(7)=-2.3422 p=0.05125	t(6)=-1.3705 p=0.2133

n=5

* Statistically significant where p<0.05

S2C Table. Two sample student t-tests were conducted to compare the cell circularity of xyloside treated cells to the no treatment control cells

Concentration / Xyloside	1 μM	10 μM	100 μM
**2**	t(5)=1.1334 p=0.3028	t(7)=0.87519 p=0.4081	t(6)=-0.031433 p=0.9758
**3**	t(7)=1.8895 p=0.0973	t(5)=0.82055 p=0.4449	t(7)=0.95736 p=0.3669
**4**	t(7)=2.9096 p=0.0205*	t(5)=0.63698 p=0.5508	t(7)=0.91502 p=0.3877

n=5

* Statistically significant where p<0.05

S2D Table. One-way ANOVA and post-hoc Tukey's test analysis of cell migration rate (per hour) in the first 12 hours of the scratch assay.

One-way ANOVA	F(9,20)=7.8704, p<0.0001***			
Post-hoc Tukey's comparisons	Mean difference	Std error	P-value	95% CL
1 µM **2** vs C	0.0757231	1.1725	0.9971	-0.2477 to 0.39915
1 µM **3** vs C	0.0209891	0.325	1	-0.30244 to 0.34442
1 µM **4** vs C*	0.387035	5.9928	0.0115	0.063609 to 0.71046
1 µM **2** vs 1 µM **3**	0.0967122	1.4975	0.9836	-0.22671 to 0.42014
1 µM **2** vs 1 µM **4****	0.462758	7.1653	0.0019	0.13933 to 0.78618
1 µM **3** vs 1 µM **4***	0.366046	5.6678	0.0189	0.04262 to 0.68947
10 µM **2** vs C	0.0867711	1.3436	0.9922	-0.23665 to 0.4102
10 µM **3** vs C	0.0976837	1.5125	0.9825	-0.22574 to 0.42111
10 µM **4** vs C	0.152321	2.3585	0.8002	-0.17111 to 0.47575
10 µM **2** vs 10 µM **3**	0.184455	2.8561	0.5964	-0.13897 to 0.50788
10 µM **2** vs 10 µM **4**	0.0655497	1.015	0.999	-0.25788 to 0.38898
10 µM **3** vs 10 µM **4**	0.250005	3.871	0.2239	-0.073421 to 0.57343
100 µM **2** vs C	0.275909	4.2721	0.1367	-0.047517 to 0.59934
100 µM **3** vs C	0.113614	1.7592	0.9553	-0.20981 to 0.43704
100 µM **4** vs C	0.294469	4.5595	0.0935	-0.028957 to 0.61789
100 µM **2** vs 100 µM **3**	0.162295	2.513	0.7413	-0.16113 to 0.48572
100 µM **2** vs 100 µM **4**	0.0185599	0.2874	1	-0.30487 to 0.34199
100 µM **3** vs 100 µM **4**	0.180855	2.8003	0.6206	-0.14257 to 0.50428
1 µM **2** vs 10 µM **2**	0.011048	0.1711	1	-0.31238 to 0.33447
1 µM **2** vs 100 µM **2***	0.351632	5.4446	0.0264	0.028206 to 0.67506
10 µM **2** vs 100 µM **2***	0.36268	5.6157	0.0204	0.039254 to 0.68611
1 µM **3** vs 10 µM **3**	0.0766946	1.1875	0.9968	-0.24673 to 0.40012
1 µM **3** vs 100 µM **3**	0.0926251	1.4342	0.9877	-0.2308 to 0.41605
10 µM **3** vs 100 µM **3**	0.0159305	0.2467	1	-0.3075 to 0.33936
1 µM **4** vs 10 µM **4*****	0.539356	8.3513	0.0003	0.21593 to 0.86278
1 µM **4** vs 100 µM **4**	0.0925664	1.4333	0.9878	-0.23086 to 0.41599
10 µM **4** vs 100 µM **4****	0.44679	6.918	0.0028	0.12336 to 0.77022

n=3

* Statistically significant where p<0.05

** Statistically significant where p<0.005

*** Statistically significant where p<0005

S2E Table. One-way ANOVA and post-hoc Tukey's test analysis of cell migration rate (per hour) from the 12th to the 30th hour of the scratch assay.

One-way ANOVA	F(9,20)= 7.3817545, p=0.00011***			
Post-hoc Tukey's comparisons	Mean difference	Std error	P-value	95% CL
1 µM **2** vs C	0.0515991	0.668	1	-0.33525 to 0.43844
1 µM **3** vs C	0.0803503	1.0402	0.9988	-0.30649 to 0.46719
1 µM **4** vs C	0.145382	1.882	0.9342	-0.24146 to 0.53223
1 µM **2** vs 1 µM **3**	0.0287512	0.3722	1	-0.35809 to 0.4156
1 µM **2** vs 1 µM **4**	0.196981	2.55	0.7263	-0.18986 to 0.58383
1 µM **3** vs 1 µM **4**	0.225732	2.9222	0.5678	-0.16111 to 0.61258
10 µM **2** vs C	0.0142746	0.1848	1	-0.37257 to 0.40112
10 µM **3** vs C	0.0840112	1.0876	0.9983	-0.30283 to 0.47086
10 µM **4** vs C	10 uM 4 vs C	0.271142	3.5101	0.3336
10 µM **2** vs 10 µM **3**	0.0982859	1.2724	0.9947	-0.28856 to 0.48513
10 µM **2** vs 10 µM **4**	0.285417	3.6949	0.2736	-0.10143 to 0.67226
10 µM **3** vs 10 µM **4**	0.187131	2.4225	0.7765	-0.19971 to 0.57398
100 µM **2** vs C	0.31756	4.111	0.1676	-0.069285 to 0.7044
100 µM **3** vs C	0.36933	4.7812	0.0689	-0.017514 to 0.75617
100 µM **4** vs C*	0.414078	5.3604	0.0299	0.027234 to 0.80092
100 µM **2** vs 100 µM **3**	0.0517704	0.6702	1	-0.33507 to 0.43861
100 µM **2** vs 100 µM **4**	0.0965186	1.2495	0.9953	-0.29033 to 0.48336
100 µM **3** vs 100 µM **4**	0.0447482	0.5793	1	-0.3421 to 0.43159
1 µM **2** vs 10 µM **2**	0.0658737	0.8528	0.9998	-0.32097 to 0.45272
1 µM **2** vs 100 µM **2**	0.369159	4.7789	0.0691	-0.017686 to 0.756
10 µM **2** vs 100 µM **2**	0.303285	3.9262	0.2098	-0.083559 to 0.69013
1 µM **3** vs 10 µM **3**	0.00628388	0.17	1	-0.17884 to 0.19141
1 µM **3** vs 100 µM **3***	0.44968	5.8213	0.015	0.062836 to 0.83652
10 µM **3** vs 100 µM **3***	0.453341	5.8687	0.0139	0.066497 to 0.84019
1 µM **4** vs 10 µM **4***	0.416524	5.3921	0.0285	0.02968 to 0.80337
1 µM **4** vs 100 µM **4**	0.268696	3.4784	0.3446	-0.11815 to 0.65554
10 µM **4** vs 100 µM **4*****	0.685221	8.8705	0.0001	0.29838 to 1.0721

n=3

* Statistically significant where p<0.05

*** Statistically significant where p<0.005

S2F Table. One-way ANOVA and post-hoc Tukey's test analysis of cell migration rate (per hour) from the 0 to the 30th hour of the scratch assay.

One-way ANOVA	F(9,20)=11.258473, p<0.0001***			
Post-hoc Tukey's comparisons	Mean difference	Std error	P-value	95% CL
1 µM **2** vs C	0.0516647	0.7674	0.9999	-0.2855 to 0.38883
1 µM **3** vs C	0.0574475	0.8533	0.9998	-0.27971 to 0.39461
1 µM **4** vs C	0.257866	3.8301	0.2348	-0.079295 to 0.59503
1 µM **2** vs 1 µM **3**	0.0057828	0.0859	1	-0.33138 to 0.34294
1 µM **2** vs 1 µM **4**	0.30953	4.5975	0.0888	-0.027631 to 0.64669
1 µM **3** vs 1 µM **4**	0.315313	4.6834	0.0789	-0.021848 to 0.65247
10 µM **2** vs C	0.0079544	0.1181	1	-0.32921 to 0.34512
10 µM **3** vs C	0.035034	0.5204	1	-0.30213 to 0.37219
10 µM **4** vs C	0.25348	3.765	0.253	-0.083681 to 0.59064
10 µM **2** vs 10 µM **3**	0.0270796	0.4022	1	-0.31008 to 0.36424
10 µM **2** vs 10 µM **4**	0.245526	3.6468	0.2884	-0.091635 to 0.58269
10 µM **3** vs 10 µM **4**	0.218446	3.2446	0.4327	-0.11871 to 0.55561
100 µM **2** vs C	0.335838	4.9882	0.0514	-0.0013224 to 0.673
100 µM **3** vs C	0.332538	4.9392	0.0552	-0.0046224 to 0.6697
100 µM **4** vs C*	0.435028	6.4615	0.0056	0.097867 to 0.77219
100 µM **2** vs 100 µM **3**	0.0033	0.049	1	-0.33386 to 0.34046
100 µM **2** vs 100 µM **4**	0.0991892	1.4733	0.9853	-0.23797 to 0.43635
100 µM **3** vs 100 µM **4**	0.102489	1.5223	0.9818	-0.23467 to 0.43965
1 µM **2** vs 10 µM **2**	0.0437103	0.6492	1	-0.29345 to 0.38087
1 µM **2** vs 100 µM **2**	0.387503	5.7556	0.0166	0.050342 to 0.72466
10 µM **2** vs 100 µM **2**	0.343793	5.1064	0.0434	0.006632 to 0.68095
1 µM **3** vs 10 µM **3**	0.0224135	0.3329	1	-0.31475 to 0.35957
1 µM **3** vs 100 µM **3***	0.389986	5.7925	0.0157	0.052825 to 0.72715
10 µM **3** vs 100 µM **3***	0.367573	5.4596	0.0258	0.030412 to 0.70473
1 µM **4** vs 10 µM **4****	0.511346	7.5951	0.001	0.17418 to 0.84851
1 µM **4** vs 100 µM **4**	0.177162	2.6314	0.6927	-0.16 to 0.51432
10 µM **4** vs 100 µM **4*****	0.688508	10.2265	< .0001	0.35135 to 1.0257

n=3
* Statistically significant where p<0.05
** Statistically significant where p<0.005
*** Statistically significant where p<0.005
