# Supplementary material for: A glycan-based approach to therapeutic angiogenesis
Source: PLoS One. 2017 Aug 1;12(8):e0182301. doi: 10.1371/journal.pone.0182301 (PMC5538652; doi:10.1371/journal.pone.0182301)
Supplement: S3 Table — Priming activity of xyloside 2, 3, and 4, and characteristics of the formed networks from the in vitro matrigel assays. (PDF) [file pone.0182301.s003.pdf]

S3 Table. Statistical analysis of the data presented in Fig 3: Priming activity of xyloside 2,3, and 4, and characteristics of the formed networks from the *in vitro* matrigel assays.

S3A Table. Fold change of the priming activity of xyloside 2, 3, and 4 relative to the no treatment control was tested with single group t-test against the mean value of 1.

Concentration / Xyloside	1 μM (n=3)	10 μM (n=3)	100 μM (n=3)
**2**	-	-	t(2)=0.95502 p=0.4404
**3**	t(2)=1.2535 p=0.3367	t(2)=3.6565 p=0.06733	t(2)=1.9139 p=0.1957
**4**	t(2)=1.4484 p=0.2845	t(2)=2.2606 p=0.1522	t(2)=2.9247 p=0.09973

S3B Table. One-way ANOVA comparing the formed networks on the matrigel from cells treated with xyloside 2, 3, 4, at 1, 10, or 100 μM concentrations, and the untreated control.

Concentration / Quantified characteristic	1 μM (n=4)	10 μM (n=4)#	100 μM (n=9)
Junctions	F(3,12)=3.6496, p=0.04446*	F(3,12)=1.3648, p=0.30046	F(3,32)=7.1985, p=0.0008**
Segments	F(3,12)=3.5261, p=0.04869*	F(3,12)=1.8831, p=0.18626	F(3,32)=7.2494, p=0.00076**
Meshes	F(3,12)=2.3076, p=0.12842	F(3,12)=1.7302, p=0.21391	F(3,32)=9.8329, p<0.0001***
Branching length	F(3,12)=1.8460, p=0.19259	F(3,12)=1.3070, p=0.31739	F(3,32)=5.9437 p=0.00243**

* Statistically significant where p<0.05
** Statistically significant where p<0.005
*** Statistically significant where p<0005
# No post-hoc test was performed, as data was not statistically significant.

S3C Table. Post-hoc Tukey's test analysis of the formed networks on the matrigel from cells treated with xylosides 2, 3, 4 at 1 μM, and the untreated control.

	Comparison	Mean difference	|q|	P-value	95% CL
Junctions	**2** vs C	0.11305	1.5081	0.7154	-0.20169 to 0.42779
	**3** vs C*	0.341117	4.5505	0.0315	0.026373 to 0.65586
	**4** vs C	0.190381	2.5397	0.3217	-0.12436 to 0.50512
	**2** vs **4**	0.0773305	1.0316	0.8834	-0.23741 to 0.39207
	**2** vs **3**	0.228067	3.0424	0.1923	-0.086677 to 0.54281
	**3** vs **4**	0.150736	2.0108	0.5103	-0.16401 to 0.46548
Segments	**2** vs C	0.109418	1.4186	0.7506	-0.21444 to 0.43327
	**3** vs C*	0.341622	4.429	0.0377	-0.017768 to 0.66548
	**4** vs C	0.202866	2.6301	0.2946	-0.12099 to 0.52672
	**2** vs **4**	0.0934477	1.2115	0.8266	-0.23041 to 0.4173
	**2** vs **3**	0.232204	3.0104	0.199	-0.09165 to 0.55606
	**3** vs **4**	0.1138756	1.7989	0.5963	-0.1851 to 0.46261
Meshes	**2** vs C	0.0813459	1.0317	0.8834	-0.2497 to 0.41239
	**3** vs C	0.284766	3.6117	0.1011	-0.046276 to 0.61581
	**4** vs C	0.12493	1.5845	0.6845	-0.20611 to 0.45597
	**2** vs **4**	0.04435841	0.5528	0.9788	-0.28746 to 0.37463
	**2** vs **3**	0.20342	2.58	0.3094	-0.12762 to 0.53446
	**3** vs **4**	0.159836	2.0272	0.5038	-0.16121 to 0.49088
Branching length	**2** vs C	0.232864	2.2947	0.4031	-0.19321 to 0.65894
	**3** vs C	0.311384	3.0684	0.1869	-0.11469 to 0.73746
	**4** vs C	0.259568	2.5578	0.3161	-0.16651 to 0.68565
	**2** vs **4**	0.0267041	0.2631	0.9976	-0.39937 to 0.45278
	**2** vs **3**	0.0785202	0.7738	0.9456	-0.34756 to 0.5046
	**3** vs **4**	0.0518161	0.5106	0.9831	-0.37426 to 0.47789

n=4

* Statistically significant where p<0.05

S3D Table. Post-hoc Tukey's test analysis of the formed networks on the matrigel from cells treated with xylosides 2, 3, 4 at 100 μM, and the untreated control.

	Comparison	Mean difference	|q|	P-value	95% CL
Junctions	2 vs C**	0.38719	5.6132	0.0021	0.12289 to 0.65149
	3 vs C	0.235247	3.4104	0.0951	-0.029051 to 0.49954
	4 vs C	0.0.0190218	0.2758	0.9973	-0.24528 to 0.28332
	2 vs 4**	0.368168	5.3374	0.0035	0.10387 to 0.63247
	2 vs 3	0.151943	2.2028	0.4164	-0.11236to 0.41624
	3 vs 4	0.216225	0.1404	0.4002	-0.048073 to 0.48052
Segments	2 vs C**	0.371205	5.6533	0.0019	0.11962 to 0.62279
	3 vs C	0.215301	3.279	0.1149	-0.036289 to 0.46689
	4 vs C	0.0155696	0.2371	0.9983	-0.23602 to 0.26716
	2 vs 4**	0.355635	5.4162	0.003	0.10405 to 0.60722
	2 vs 3	0.155904	2.3744	0.3511	-0.095686 to 0.40749
	3 vs 4	0.199732	3.0418	0.1591	-0.051858 to 0.45132
Meshes	2 vs C***	0.366714	6.7902	0.0002	0.15978 to 0.57365
	3 vs C*	0.20694	3.8318	0.05	8.2746e-06 to 0.41387
	4 vs C	0.0344673	0.6382	0.9689	-0.17246 to 0.2414
	2 vs 4**	0.332247	6.152	0.0007	0.12531 to 0.53918
	2 vs 3	0.159774	2.9584	0.1774	-0.047157 to 0.36671
	3 vs 4	0.172472	3.1936	0.1295	-0.034459 to 0.3794
Branching length	2 vs C**	0.406091	5.4399	0.0029	0.12006 to 0.69212
	3 vs C	0.275199	3.6865	0.0628	-0.010831 to 0.56123
	4 vs C	0.0932285	1.2489	0.8136	-0.1928 to 0.37926
	2 vs 4*	0.312863	4.1911	0.0277	0.026832 to 0.59889
	2 vs 3	0.130892	1.7534	0.6068	-0.15514 to 0.41692
	3 vs 4	0.181971	2.4376	0.3285	-0.10406 to 0.468

n=9

* Statistically significant where p<0.05

** Statistically significant where p<0.005

*** Statistically significant where p<0005

S3E Table. Fold change of the formed network characteristics from cells treated with xylosides 2, 3, 4 at 100 µM relative to the no treatment control was tested with single group t-test against the mean value of 1.

Concentration	Xyloside	Junctions	Segments	Meshes	Branching length
1 µM (n=4)	**2**	t(3)=1.9786 p=0.1423	t(3)=1.6571 p=0.1961	t(3)=0.94395 p=0.4148	t(3)=1.8942 p=0.1545
	**3**	t(3)=5.8021 p=0.01019*	t(3)=6.0075 p=0.00924*	t(3)=6.2297 p=0.00834*	t(3)=6.586 p=0.007123*
	**4**	t(3)=1.8162 p=0.167	t(3)=1.931 p=0.149	t(3)=1244 p=0.3018	t(3)=2.1234 p=0.1238
10 µM (n=4)	**2**	t(3)=1.7737 p=0.1742	t(3)=1.772 p=0.1745	t(3)=1.6411 p=0.1993	t(3)=2.7331 p=0.07175
	**3**	t(3)=1.7056 p=0.1866	t(3)=2.5191 p=0.08625	t(3)=2.7591 p=0.0702	t(3)=1.7565 p=0.1773
	**4**	t(3)=2.2145 p=0.1136	t(3)=2.388 p=0.09692	t(3)=2.3572 p=0.09965	t(3)=2.4079 p=0.0952
100 µM (n=9)	**2**	t(8)=5.2575 p=0.0007668**	t(8)=5.7679 p=0.0004203***	t(8)=7.1811 p<0.0001***	t(8)=7.5608 p<0.0001***
	**3**	t(8)=3.5961 p=0.007022*	t(8)=3.3617 p=0.009906*	t(8)=3.3276 p=0.01042*	t(8)=3.9892 p=0.00401**
	**4**	t(8)=0.29758 p=0.7736	t(8)=0.26582 p=0.7971	t(8)=0.66836 p=0.5227	t(8)=1.0446 p=0.3267

* Statistically significant where p<0.05
** Statistically significant where p<0.005
*** Statistically significant where p<0005

S3F Table. One-way ANOVA and post-hoc Tukey's test of the formed networks on the matrigel from cells treated with conditioned media of cells primed with xylosides 2, 3, 4 at 100 µM, and the untreated control.

One-way ANOVA	Post-hoc Tukey's Comparisons	Mean difference	Std error	P-value	95% CL
Junctions F(3,8)=3.9363, p=0.0538	**2** vs C	0.287074	2.3948	0.386	-0.25582 to 0.82997
	**3** vs C	0.536168	4.4727	0.0529	-0.0067239 to 1.0791
	**4** vs C	0.0892367	0.7444	0.9503	-0.45366 to 0.63213
	**2** vs **4**	0.197837	1.6504	0.6621	-0.34506 to 0.74073
	**2** vs **3**	0.24905	2.078	0.496	-0.29380 to 0.79199
	**3** vs **4**	0.446932	3.7283	0.1111	-0.095961 to 0.98982
Segments F(3,8)=6.8691, p=0.01325*	**2** vs C	0.464962	4.1826	0.0706	-0.038484 to 0.96841
	**3** vs C*	0.673411	6.0578	0.0114	0.16997 to 1.1769
	**4** vs C	0.229113	2.061	0.5023	-0.27433 to 0.73256
	**2** vs **4**	0.235849	2.1216	0.4799	-0.26760 to 0.73929
	**2** vs **3**	0.20845	1.8751	0.5734	-0.29500 to 0.71190
	**3** vs **4**	0.444299	3.9967	0.085	-0.059147 to 0.94774
Meshes F(3,8)=4.2314, p=0.04563*	**2** vs C	0.444831	3.3423	0.1624	-0.15791 to 1.0476
	**3** vs C*	0.62963	4.7308	0.0409	0.026887 to 1.2324
	**4** vs C	0.215008	1.6155	0.6759	-0.38773 to 0.81775
	**2** vs **4**	0.229823	1.7268	0.6318	-0.37292 to 0.83257
	**2** vs **3**	0.184799	1.3885	0.7635	-0.41794 to 0.78754
	**3** vs **4**	0.414622	3.1153	0.202	-0.18812 to 1.0174
Branching length F(3,8)=9.2022, p=0.00568*	**2** vs C*	0.486085	5.1462	0.0272	0.058319 to 0.91385
	**3** vs C**	0.673039	7.1255	0.0044	0.24527 to 1.1008
	**4** vs C	0.305612	3.2356	0.18	-0.12215 to 0.73338
	**2** vs **4**	0.180473	1.9107	0.5595	-0.24729 to 0.60824
	**2** vs **3**	0.186954	1.9793	0.5331	-0.24081 to 0.61472
	**3** vs **4**	0.367427	3.89	0.0946	-0.06034 to 0.79519

n=3
* Statistically significant where p<0.05
** Statistically significant where p<0.005
*** Statistically significant where p<0005

S3G Table. Fold change of the formed network characteristics from cells treated conditioned media of cells primed with xylosides 2, 3, 4 at 100 µM relative to the no treatment control was tested with single group t-test against the mean value of 1.

Xyloside	Junctions	Segments	Meshes	Branching length
**2**	t(2)=2.4126 p=0.1373	t(2)=8.7266 p=0.01288	t(2)=7.4111 p=0.01772*	t(2)=3.7943 p=0.06297
**3**	t(2)=35.781 p=0.0007802**	t(2)=23.453 p=0.001813**	t(2)=20.526 p=0.002365**	t(2)=17.919 p=0.0031**
**4**	t(2)=0.47412 p=0.6821	t(2)=1.1197 p=0.3793	t(2)=0.95427 p=0.4407	t(2)=2.3548 p=0.1427

n=3
* Statistically significant where p<0.05
** Statistically significant where p<0.005
*** Statistically significant where p<0005
