# Supplementary material for: A glycan-based approach to therapeutic angiogenesis
Source: PLoS One. 2017 Aug 1;12(8):e0182301. doi: 10.1371/journal.pone.0182301 (PMC5538652; doi:10.1371/journal.pone.0182301)
Supplement: S4 Table — P-ERK1/2 / ERK1/2 expression of cells treated with xyloside 3 and 4 at 100 μM, and the untreated control (western blot densitometry data). (PDF) [file pone.0182301.s004.pdf]

S4 Table. Statistical analysis of the data presented in Fig 6; P-ERK1/2 / ERK1/2 expression of cells treated with xyloside 3 and 4 at 100 µM, and the untreated control (western blot densitometry data).

One-way ANOVA	F(2,9)=7.327874, p=0.01292			
Post-hoc Tukey's comparisons	Mean difference	Std error	P-value	95% CL
**3** vs C	0.176142	3.7421	0.0628	-0.0097151 to 0.362
**4** vs C*	0.247564	5.2594	0.012	0.061707 to 0.43342
Two-sample student's t-test				
**3** vs C*	t(4)=-3.4176, p=0.02543			
**4** vs C*	t(3)=-3.7203, p=0.02407			

n=4

* Statistically significant where p<0.05
