# Supplementary material for: A glycan-based approach to therapeutic angiogenesis
Source: PLoS One. 2017 Aug 1;12(8):e0182301. doi: 10.1371/journal.pone.0182301 (PMC5538652; doi:10.1371/journal.pone.0182301)
Supplement: S5 Table — Quantified VEGFR1 fluorescence from the cryosections of the xyloside 3 loaded collagen gels, placed on the chick chorioallantoic membrane. (PDF) [file pone.0182301.s005.pdf]

S5 Table. Statistical analysis of the data presented in Fig 7; Quantified VEGFR1 fluorescence from the cryosections of the xyloside 3 loaded collagen gels, placed on the chick chorioallantoic membrane.

Fold change relative to control (single group t-test against the mean value of 1)	
**Treatment** vs C*	t(4)=3.2228, p=0.03219

For control gels, gels loaded with 1 mM, and 10 mM xyloside **3**, n=3, for 5 mM, n=2.
As the sample size for the 5 mM concentration was too small for statistical analysis, the values for 1 mM and 5 mM was grouped together as xyloside treated group
* Statistically significant where $p<0.05$
