# Supplementary material for: A glycan-based approach to therapeutic angiogenesis
Source: PLoS One. 2017 Aug 1;12(8):e0182301. doi: 10.1371/journal.pone.0182301 (PMC5538652; doi:10.1371/journal.pone.0182301)

S1 Fig. VEGFR1 positive cells coincide with VEGFR2 positive cells. Cross sections of the collagen gel on the chick chorioallantoic membrane were immunofluorescently stained with antibodies against VEGFR1 (Green) and VEGFR2 (Red), and DAPI (Blue) for nuclear staining. The staining appears yellow where VEGFR1 and VEGFR2 staining coincides. The arrows indicate lumen formation.

S1A Fig. High magnification images of the cross section of the control collagen gels.

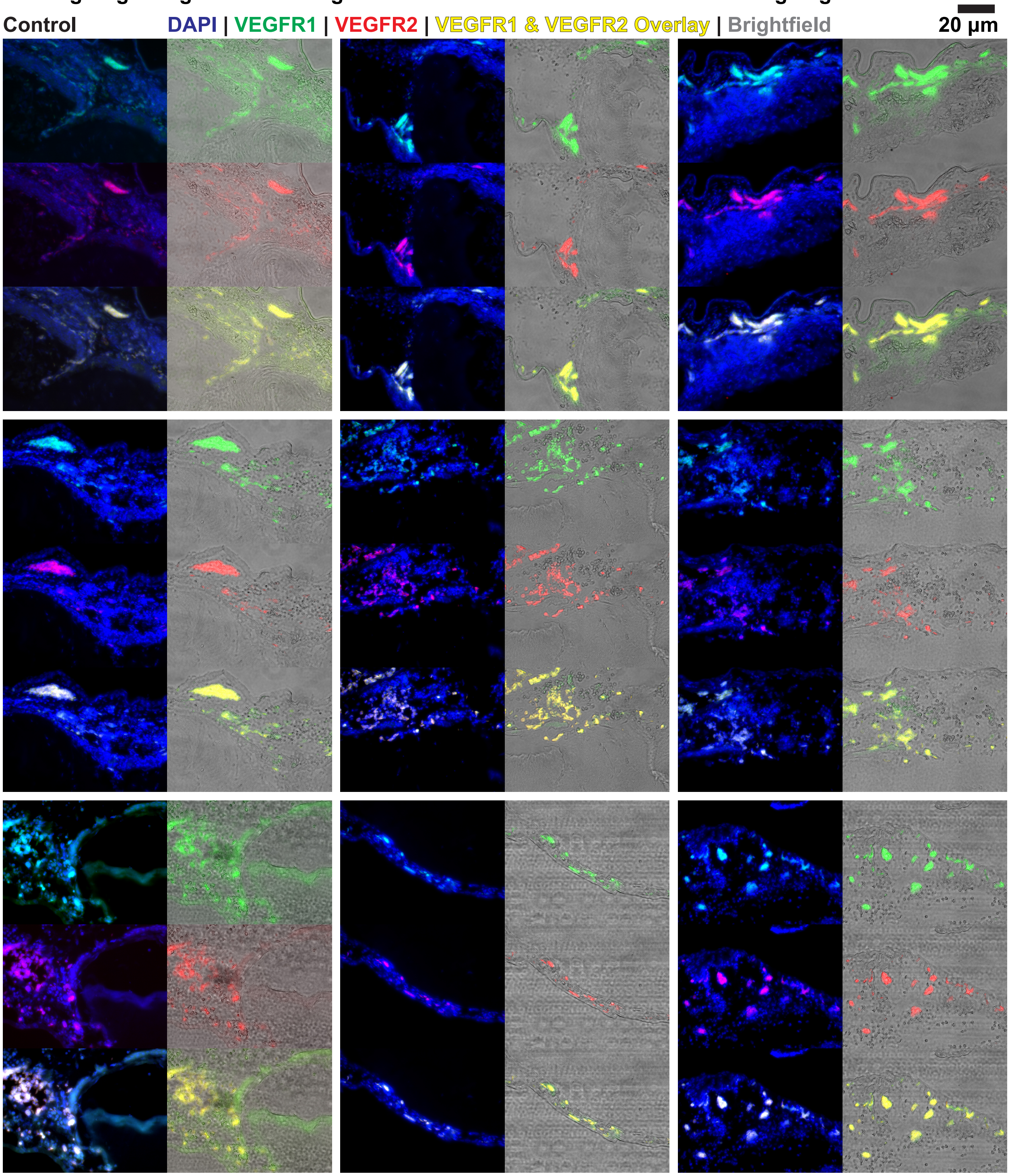

S1B Fig. High magnification images of the cross section of the collagen gels loaded with 1 mM xyloside 3.

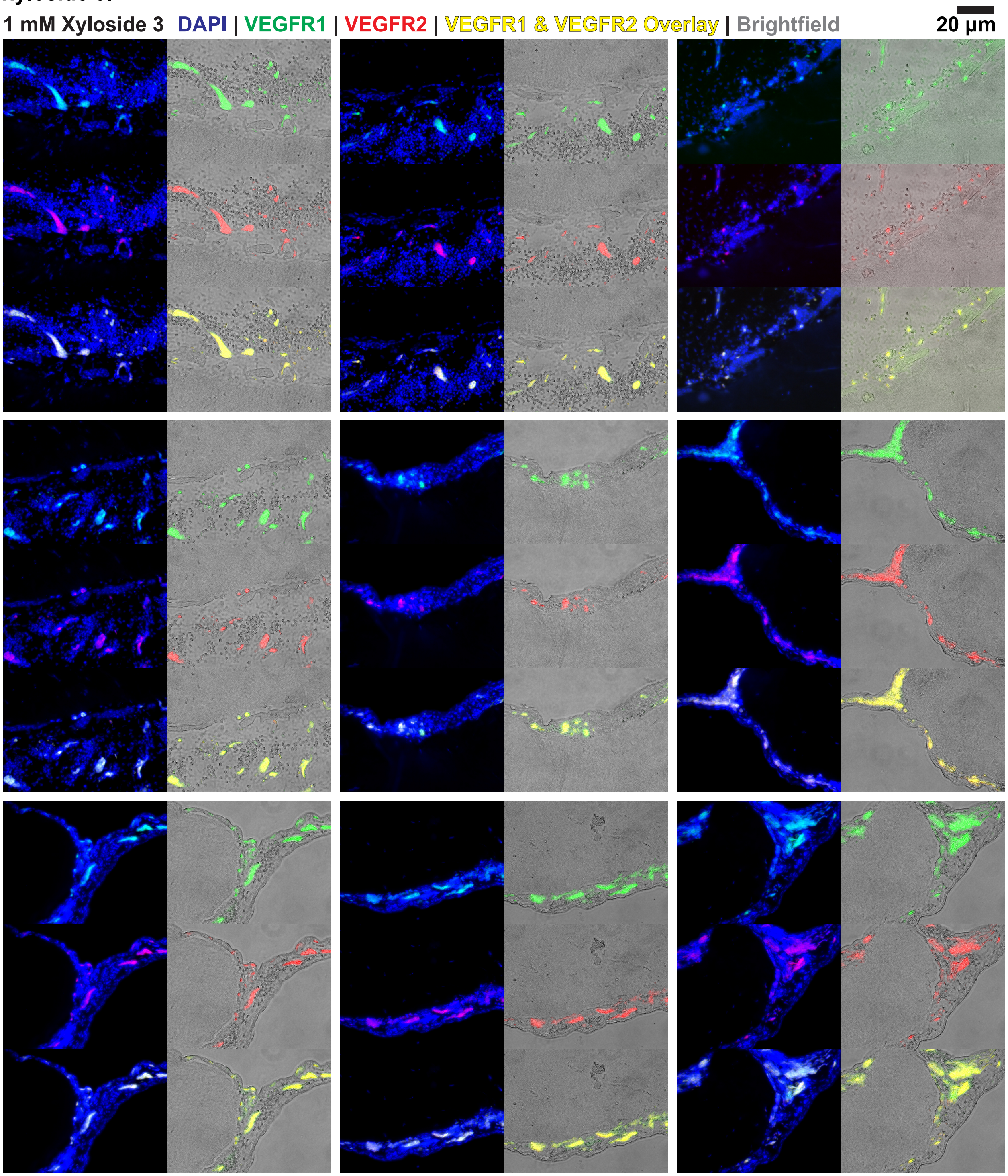

S1C Fig. High magnification images of the cross section of the collagen gels loaded with 5 mM xyloside 3.

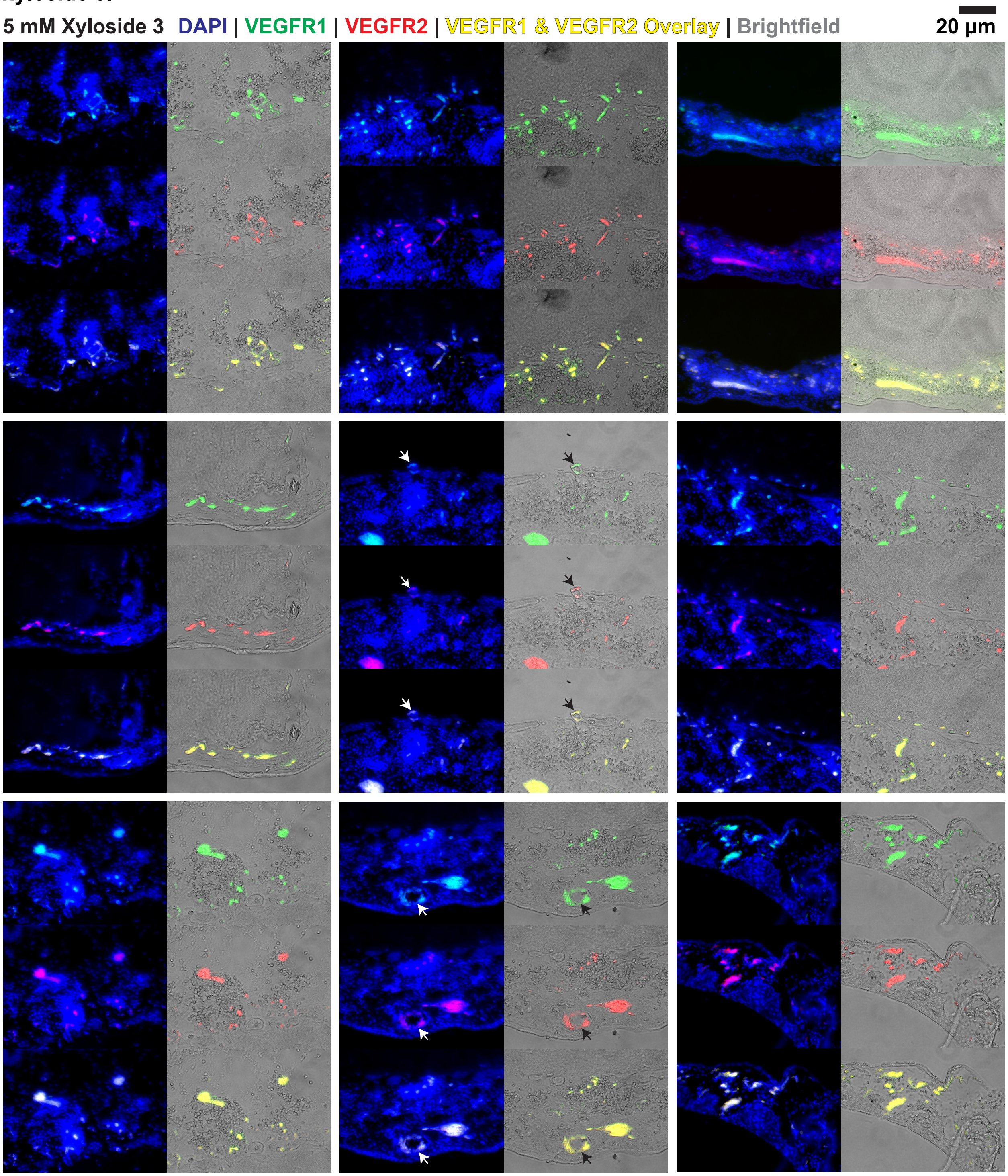

S1D Fig. High magnification images of the cross section of the collagen gels loaded with 10 mM xyloside 3.

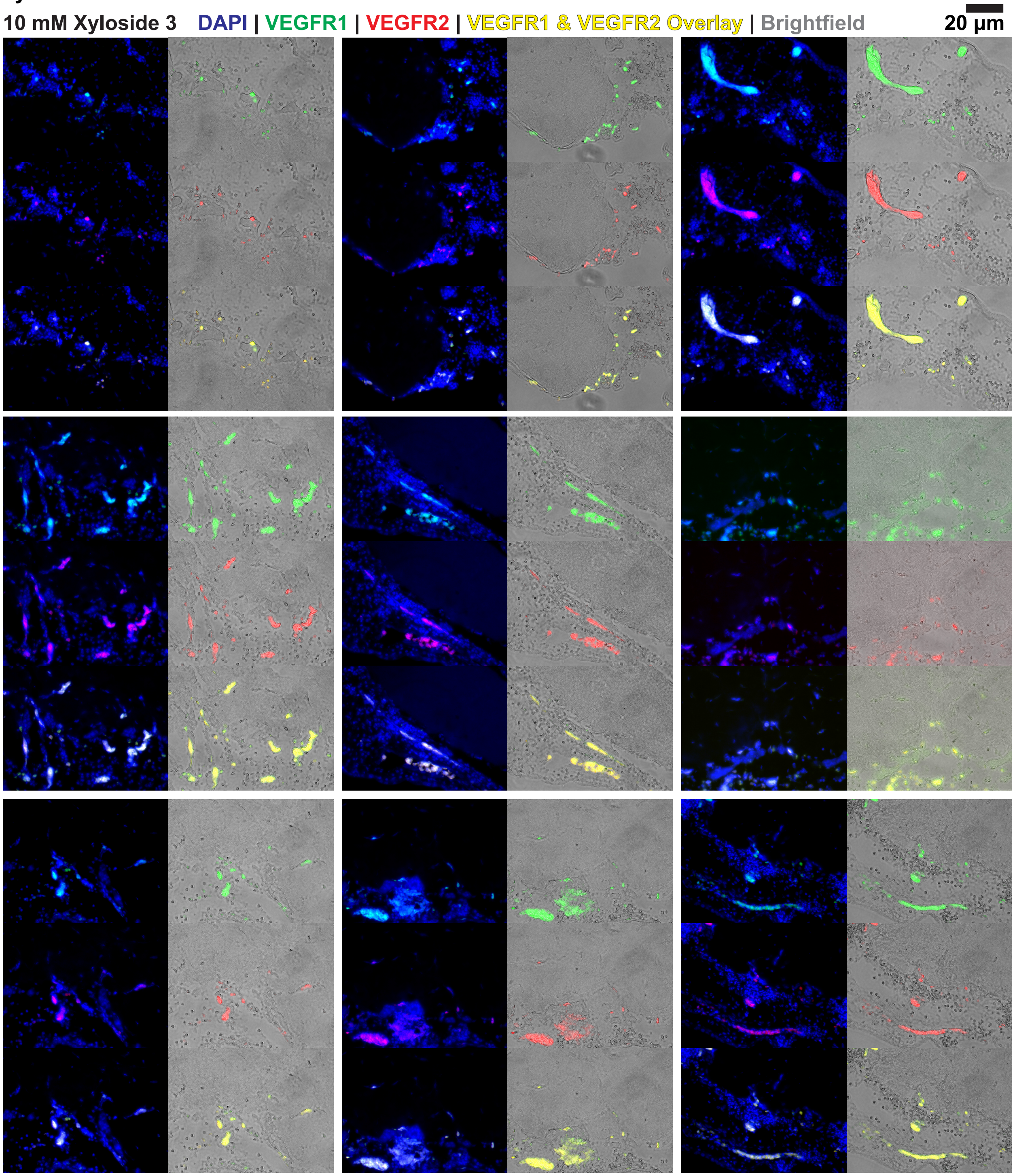

Supplement: S1 Fig — Cross sections of the collagen gel on the chick chorioallantoic membrane were immunofluorescently stained with antibodies against VEGFR1 (Green) and VEGFR2 (Red), and DAPI (Blue) for nuclear staining. The staining appears yellow where VEGFR1 and VEGFR2 staining coincides. The arrows indicate lumen formation. (PDF) [file pone.0182301.s006.pdf]
